# Placental expanded mesenchymal-like cells (PLX-R18) for poor graft function after hematopoietic cell transplantation: A phase I study

**DOI:** 10.1038/s41409-023-02068-3

**Published:** 2023-08-08

**Authors:** Joseph P. McGuirk, Leland Metheny, Luis Pineiro, Mark Litzow, Scott D. Rowley, Batia Avni, Roni Tamari, Hillard M. Lazarus, Jacob M. Rowe, Michal Sheleg, Daniel Rothenstein, Nitsan Halevy, Tsila Zuckerman

**Affiliations:** 1 Division of Hematologic Malignancies & Cellular Therapeutics, University of Kansas Medical Center, Kansas City, KS USA; 2 Case Western Reserve University, Cleveland, OH USA; 3 University Hospitals Seidman Cancer Center, Cleveland, OH USA; 4 Apheresis and Marrow Processing Laboratories, Baylor University Medical Center, Dallas, TX USA; 5 Division of Hematology, Mayo Clinic, Rochester, MN USA; 6 Stem Cell Transplantation and Cellular Therapy Program, John Theurer Cancer Center, Hackensack, NJ USA; 7 Hadassah University Medical Center, Jerusalem, Israel; 8 Memorial Sloan Kettering Cancer Center, New York, NY USA; 9 Case Western Reserve University School of Medicine, Cleveland, OH USA; 10 Department of Hematology, Shaare Zedek Medical Center, Jerusalem, Israel; 11 The Ruth and Bruce Rappaport Faculty of Medicine, Technion, Haifa, Israel; 12 Department of Hematology and Bone Marrow Transplantation, Rambam Health Care Campus, Haifa, Israel; 13 Pluri-Biotech Ltd., Haifa, Israel

**Keywords:** Drug development, Haematopoietic stem cells, Haematological diseases, Therapeutics, Bone marrow transplantation

## Abstract

Persistent cytopenia in the post-hematopoietic cell transplantation (HCT) setting can occur despite adequate engraftment of donor cells. PLX-R18, a placental-derived mesenchymal-like cell product, is expanded ex vivo in a 3-dimensional environment. PLX-R18 cells secrete a large array of hematopoietic factors, which promote regeneration, maturation, and differentiation of hematopoietic cells and stimulate their migration to peripheral blood. This phase 1, first-in-human study (NCT03002519), included 21 patients with incomplete hematopoietic recovery post-HCT. Patients were treated with escalating doses of PLX-R18: 3 patients received 1 million cells/kg, 6 received 2 million cells/kg, and 12 received 4 million cells/kg via multiple intramuscular injections. While patients received only two administrations of cells during the first week, peripheral blood counts continued to increase for months, peaking at 6 months for hemoglobin (Hb, *p* = 0.002), lymphocytes (*p* = 0.008), and neutrophils (ANC, *p* = 0.063), and at 9 months for platelets (*p* < 0.001) and was maintained until 12 months for all but ANC. The need for platelet transfusions was reduced from 5.09 units/month at baseline to 0.55 at month 12 (*p* = 0.05). Likewise, red blood cell transfusions decreased from 2.91 units/month at baseline to 0 at month 12 (*p* = 0.0005). PLX-R18 was safe and well tolerated and shows promise in improving incomplete hematopoietic recovery post-HCT.

## Introduction

The American Society for Transplantation and Cellular Therapy 2021 guidelines [1] define clinical conditions, which manifest as low peripheral counts, in one or more cell lines, including graft failure, graft rejection, and poor graft function (PGF). PGF is defined as decreased peripheral blood counts in the presence of full donor chimerism, and in the absence of various conditions (e.g., infection, disease recurrence, certain drugs). PGFBM may result from inadequate hematopoietic stem and progenitor cell infusion, or be associated with various endogenous (i.e., fibrosis) or exogenous (i.e., damage brought on by conditioning regimen) aspects of the bone marrow (BM) microenvironment [2, 3]. PGF occurs in 5-27% of patients following allo-HCT [4–6] and ~2% following auto-HCT [7] and represents a clinically meaningful unmet need. In addition to identifying an underlying cause of cytopenia, management includes repeated blood product transfusions, hematopoietic growth factors (HGF) such as G-CSF and thrombopoietin receptor agonists (TPO RA), and a CD34+ hematopoietic cell boost [8, 9].

Mesenchymal stromal/stem cells (MSCs) have pro-hematopoietic and immunomodulatory properties and secrete various cytokines and proteins that modulate the BM microenvironment and support hematopoiesis. Clinical application of MSCs to support the hematopoietic system post-HCT have been previously undertaken to promote cell engraftment, prevent and treat GvHD, and treat PGF and aplastic anemia [10, 11]. Specifically, the use of MSCs for the treatment of patients with incomplete hematopoietic recovery/PGF post-HCT has been investigated in several studies, in which most patients demonstrated improvement [12–15]. PLX-R18 (avoplacel, Pluri-Biotech Ltd., Haifa, Israel) is being developed for the treatment of PGF post-HCT. Preliminary preclinical studies have first demonstrated the effect of the intra-muscular injection of non-matched human PLX-R18 for a fast mitigation of acute radiation syndrome in a mouse model, with highly elevated survival rate [16]. The mechanism of action of the IM injected PLX-R18 in the irradiated mice is proposed to be mediated through the PLX-R18 stress induced secretome of multiple hematopoietic factors. Those include stem cell factor (SCF), granulocyte-macrophage colony-stimulating factor (GM-CSF), granulocyte colony-stimulating factor (G-CSF), interleukin (IL)-6 and IL-11, enhance regeneration, maturation and differentiation of hematopoietic cells and the migration of mature blood cells to peripheral blood (see Table S1 of selected secreted factors). The PLX-R18 induced earlier increased endogenous secretion of murine G-CSF and MCP-1 among others, contributing to recovery of the irradiated BM [17]. Additionally, PLX-R18 cells possess immunomodulatory properties, such as inhibition of Th1 and induction of Treg and M2 macrophages [18], which make PLX-R18 a suitable candidate to address immune changes in the BM microenvironment which may play an important role in PGF pathophysiology [19, 20]. The objective of this phase 1, multicenter, open-label, dose escalation study was to determine the safety profile of IM PLX-R18 in auto- and allo-HCT recipients. This report summarizes the results of the study (NCT03002519).

## Methods

### Patients

The study was conducted from September 2017 to August 2020 and included 21 patients treated with PLX-R18 in 8 study sites in the US and Israel. Adult patients ( ≥ 18 years), at least 3 months after allo- or auto-HCT with sustained cytopenia, defined as platelet (PLT) count ≤ 50,000/µL, and/or Hb ≤ 8 g/dL, and/or ANC ≤ 1000/µL, were eligible for the study. Cytopenia was confirmed by at least 2 consecutive blood counts and a hypocellular BM. Complete and stable donor chimerism in at least 3 consecutive tests prior to treatment was required. Patients had to have no other observed causes of cytopenia (e.g., active infection, disease recurrence, grade 3–4 acute GvHD or severe chronic GvHD) at the time of study entry. Finally, patients needed to have a life expectancy of over 6 months and a score of 0–2 on the Eastern Cooperative Oncology Group (ECOG) performance scale. Complete eligibility criteria are listed in the supplementary information- List S2. This study was conducted in adherence to good clinical practices and approved by relevant institutional review boards (See complete list of ethics committees/institutional review boards in Table S17) and country regulatory authorities. All patients gave written informed consent.

### Study Treatment

PLX-R18 is an allogeneic ex-vivo placental expanded adherent stromal cell product derived from full-term human placentae of healthy women undergoing an elective caesarean section (see supplementary information S3 for further details). PLX-R18 cells are MSC-like, have a high expression of typical MSC markers (CD105, CD73 and CD29) and lack expression of CD45, CD34, CD14, CD19, and HLA-DR [18]. Compared to BM derived MSCs, PLX-R18 exhibit a limited capacity to differentiate in vitro into osteocytes and adipocytes [21]. PLX-R18 was provided as a frozen cell dispersion in 6 mL vials, at a concentration of either 10 or 20 million cells/mL, in a solution containing 4% DMSO (v/v) and 5% human serum albumin (w/v) in Plasma-Lyte. PLX-R18 is stored in the vapor phase of liquid nitrogen below −150 °C, thawed at the clinical site, and administered without further manipulation. In-use stability studies show PLX-R18 cells quality is maintained per the specifications post-thawing and during vial preparation until administration [22].

All patients were treated with PLX-R18 in addition to standard of care, including blood product transfusions and HGF, as deemed clinically necessary by the investigators. The first 3 patients were assigned to Cohort 1 (1 million cells/kg/administration), the next 6 patients to Cohort 2 (2 million cells/kg/administration) and the last 12 patients to Cohort 3 (4 million cells/kg/administration). Each patient received 2 administrations of PLX-R18, 7 days apart. Individual dose and number of injections was calculated for each patient based on cohort assignment, body weight, and PLX-R18 cell concentration. Maximal dose was not to exceed 400 million cells per administration session; maximal volume not to exceed 1.5 mL per injection site. The required total dose of PLX-R18 was injected in multiple intramuscular (IM) injections, preferably bilaterally, into the gluteus medius and thigh muscles. All patients were pre-treated with an antihistamine, and if desired a topical anesthetic at the planned injection sites. All patients were hospitalized for 24 h after each administration to closely monitor short term adverse events (AEs). To minimize risk, patients were treated sequentially, with a minimal pre-defined lapse (4 weeks from first dose) between cohorts, and with the oversight of an independent Data Safety Monitoring Board that assessed safety data in an ongoing manner and prior to dose escalation. The highest dose, 4 million cells/kg, is 10-fold lower than the dose proven to be safe in toxicology murine and non-human-primates (NHP) studies. NHP data further supported this dose as sufficient to demonstrate a response. The IM route of administration was selected following murine and NHP studies showing favorable persistence following IM administration [21].

### Study assessments

Patients were followed up for 12 months from the first PLX-R18 administration. Study visits occurred after PLX-R18 administration on days 0 (first PLX-R18 administration), 1, 7 (second PLX-R18 administration), 8, 14 and 28 and thereafter at month 3, 6, 9 and 12. A full schedule of assessments is included in Table S4. Briefly, assessments included a physical examination including GvHD status, vital signs, and ECG, recording all treatment emergent adverse events (TEAEs), concomitant medications, and blood transfusions. Laboratory assessment included kidney and liver function, peripheral blood counts, routine urinalysis, anti-HLA antibodies, chimerism, and EBV/CMV viral load. BM biopsies were performed prior to treatment and at 12 weeks post treatment and assessed by a central lab. The SF-36v2 questionnaire was used to assess quality of life [23].

### Statistical analysis

The primary outcome of this study was safety. Exploratory efficacy outcomes were collected and included changes from baseline in PLT, ANC and Hb, as well as changes in transfusion frequency (see supplementary information List S5 for a complete list). In some analyses, to better elucidate a potential treatment effect considering the limitation of sample size and as all patients were treated with PLX-R18, all treated patients were grouped and analyzed as a pooled cohort. Descriptive statistics were applied to all variables, including mean, standard deviation, median, minimum, and maximum. Changes from baseline were to be compared between the study doses using Mixed Models Repeated Measures (MMRM) for variables with repeated measures, or Analysis of Covariance (ANCOVA) model for variables with only one post-baseline measure.

## Results

### Patients

**Fig. 1:**
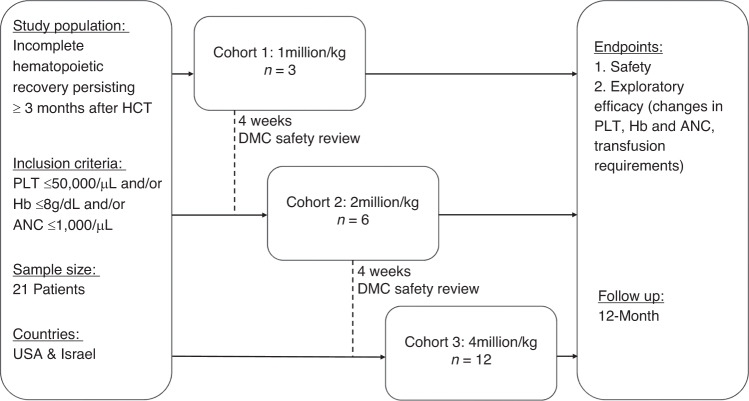
Study design.

A total of 35 patients were screened, 22 were enrolled, and 21 were treated; one patient enrolled into Cohort 2 died prior to receiving PLX-R18. For a summary of screened non-randomized patients see Table S6. The study enrolled a heterogenous population with various underlying diseases leading to HCT and a wide range of durations from HCT to study entry. Demographics and underlying disease history are presented in Table 1. Patients were treated with PLX-R18 as assigned (see “Study Treatment” in Methods section above and Fig. 1). All treated patients received 2 administrations of PLX-R18. Six of the 21 patients did not complete 12 months of follow up: 4 patients died (2 in Cohort 2 and 2 in Cohort 3), 1 patient was lost to follow up (Cohort 1), and 1 patient was discontinued due to “persistent PGF requiring additional treatment” (Cohort 3).

**Table 1:** Demographics, baseline characteristics, underlying disease and HCT history.

	PLX-R18, 1 M cells/kg (Cohort 1), *N* = 3	PLX-R18, 2 M cells/kg (Cohort 2), *N* = 6	PLX-R18, 4 M cells/kg (Cohort 3), *N* = 12	Overall, *N* = 21	*P*-value
**Demographics**					
Age, years, mean (SD)	49.0 (5.6)	57.0 (3.8)	57.0 (15.1)	55.9 (11.9)	0.58
Male, *n* (%)	2 (66.7)	2 (33.3)	8 (66.7)	12 (57.1)	0.47
Caucasian. *N* (%)	3 (100.0)	5 (83.3)	12 (100.0)	20 (95.2)	0.43
BMI, kg/m^2^, mean (SD)	33.5 (7.5)	26.0 (5.1)	29.6 (6.7)	29.1 (6.6)	0.26
**Blood count at baseline**					
Platelets, cells x 10^3^/μL, median (min, max)	24 (4.0, 24.5)	21.8 (12.0, 46.0)	36.3 (11.0, 83.25)	30.5 (4.0, 83.25)	0.06
platelets < 10 × 10^3^/μL, n(%)	1 (33.3)	0 (0.0)	0 (0.0)	1 (4.8)	0.14
platelets < 20 × 10^3^/μL, n(%)	1 (33.3)	2 (33.3)	2 (16.7)	5 (23.8)	0.64
Hemoglobin, g/dL, median (min, max)	8.7 (8.5, 8.9)	8.3 (6.5, 11.4)	9.1 (6.3, 11.8)	8.8 (6.3, 11.8)	0.61
ANC, cells x 10^3^/μL, median (min, max)	2.1 (0.9, 3.2)	1.2 (0.8, 1.6)	1.2 (0.2, 1.9)	1.3 (0.2, 3.2)	0.09
ANC < 0.5 × 10^3^/μL, n(%)	0 (0.0)	0 (0.0)	3 (25.0)	3 (14.3)	0.70
ANC < 1 × 10^3^/μL, n(%)	1 (33.3)	1 (16.7)	5 (41.7)	7 (33.3)	0.81
Lymphocytes, cells x 10^3^/μL, median (min, max)	0.4 (0.3, 1.4)	0.5 (0.1, 1.4)	0.7 (0.3, 2.3)	0.7 (0.1, 2.3)	0.76
**Disease history**					
Primary Diagnosis					
Acute lymphoblastic leukemia (ALL), *n*(%)	1 (33.3)	3 (50.0)	3 (25.0)	7 (33.3)	
Acute myelogenous leukemia (AML), *n*(%)	1 (33.3)	0 (0.0)	2 (16.7)	3 (14.3)	
Multiple myeloma, *n*(%)	0 (0.0)	0 (0.0)	2 (16.7)	2 (9.5)	
Myelodysplastic syndrome (MDS), *n*(%)	0 (0.0)	1 (16.7)	1 (8.3)	2 (9.5)	
Non-Hodgkin lymphoma (NHL), *n*(%)	1 (33.3)	0 (0.0)	1 (8.3)	2 (9.5)	
Other (malignant), *n*(%)	0 (0.0)	2 (33.3)	3 (25.0)	5 (23.8)	0.81
HCT type, Allogeneic, *n*(%)	2 (66.7)	6 (100.0)	11 (91.7)	19 (90.5)	0.34
HCT cell source					
Bone marrow, *n*(%)	1 (33.3)	1 (16.7)	4 (33.3)	6 (28.6)	
Peripheral blood, *n*(%)	1 (33.3)	5 (83.3)	7 (58.3)	13 (61.9)	
Umbilical cord, *n*(%)	1 (33.3)	0 (0.0)	1 (8.3)	2 (9.5)	0.51
Conditioning Regimen, Myeloablative *n* (%)	3 (100.0)	3 (50.0)	8 (66.7)	14 (66.7)	0.86
Time from HCT, days, median (min, max)	450 (872, 224)	188 (540, 160)	273 (792, 118)	236 (872, 118)	0.28
GVHD at screening, *n* (%)	0 (0.0)	1 (16.7)	5 (41.7)	6 (28.6)	1.00
Acute, *n*	0	0	1	1	
Chronic, *n*	0	1	4	5	
Previous HCT, *n*(%)	0 (0.0)	2 (33.3)	3 (25.0)	5 (23.8)	0.81
**Transfusion dependence and history**					
Transfusion dependence, as defined by Investigator, *n*(%)	3 (100.0)	4 (66.7)	6 (50.0)	13 (61.9)	0.34
Number of PLT transfusions per month at baseline, mean (SD)*	4.9 (2.1)	2.8 (2.4)	6.8 (10.3)	5.1 (7.1)	0.66
Number of RBC transfusions per month at baseline, mean (SD)*	3.1 (0.8)	2.6 (1.1)	3.1 (2.5)	2.9 (1.8)	0.91

*BMI* Body Mass Index, *PLT* Platelets, *HGB* Hemoglobin, *ANC* Absolute Neutrophil Count, *HCT* Hematopoietic Cell Transplantation, *GHVD* Graft Versus Host Disease, *RBC* Red Blood Cells.

*Calculated number of transfusions at baseline was calculated by adding all recorded transfusions in the 3 months prior to study entry and dividing by 3; this number was calculated for any patient who received transfusions at any time (*n* = 15).

### Safety

All patients had at least 1 TEAE (Table 2), 71% had at least one serious TEAE and 67% had at least one severe TEAE. While most patients had at least one event that was considered related (76%), only one related event was assessed as serious; a Cohort 3 patient who developed febrile neutropenia 2 days after the 2nd PLX-R18 administration and 5 months after allo-HCT for acute lymphocytic leukemia (ALL). The patient had a fever of 38.3 °C at an ANC of 0.17 × 10^9^/L, with no apparent source. The patient was treated with empiric cefepime, recovered two days later, and was discharged from the hospital with no sequelae. Two additional patients developed febrile neutropenia, 82 and 353 days following PLX-R18 administration. Both were assessed as unrelated to PLX-R18 by the Investigators.

**Table 2:** Patients with at least one TEAE by seriousness, relatedness, and severity.

	PLX-R18	PLX-R18	PLX-R18	Overall
	1 M cells/kg (Cohort 1)	2 M cells/kg (Cohort 2)	4 M cells/kg (Cohort 3)	
	*N* = 3	*N* = 6	*N* = 12	*N* = 21
	*n* (%)	*n* (%)	*n* (%)	*n* (%)
Patients with any TEAEs [1]	3 (100.0)	6 (100.0)	12 (100.0)	21 (100.0)
Patients with any serious TEAEs	3 (100.0)	4 (66.7)	8 (66.7)	15 (71.4)
Patients with TEAEs with fatal outcome	0	2 (33.3)	2 (16.7)	4 (19.0)
**Patients with TEAEs by relationship throughout the study [2]**				
Related	3 (100.0)	2 (33.3)	11 (91.7)	16 (76.2)
Not related	0	4 (66.7)	1 (8.3)	5 (23.8)
**Patients with TEAEs by highest severity throughout the study [2]**				
Mild – Grade 1	0	0	3 (25.0)	3 (14.3)
Moderate – Grade 2	0	2 (33.3)	2 (16.7)	4 (19.0)
Severe – Grade 3-5	3 (100.0)	4 (66.7)	7 (58.3)	14 (66.7)

*TEAE* Treatment emergent adverse event.

[1] All non-serious TEAEs and serious TEAEs were collected throughout the study; TEAEs include serious TEAEs; all deaths were collected throughout the study.

[2] In case of multiple events, the highest severity or relationship was counted.

One 46-year-old patient had a recurrence of the underlying malignancy (ALL), 290 days after PLX-R18 administration (Cohort 3) and 702 days after HCT and died 2 weeks later. ALL recurrence and death were assessed as unrelated to PLX-R18. Three additional patients died during follow up: a 60-year-old who died due to sepsis following pneumonia 20 days after PLX-R18 treatment (Cohort 2); a 54-year-old who died due to septic shock following infected calf injury 92 days after PLX-R18 treatment (Cohort 2); and a 63-year-old found dead at home 113 days after PLX-R18 (Cohort 3) and classified as sudden unexplained death. None of the fatal events were considered related to PLX-R18. For brief narratives of fatal events, see supplementary information S7.

The most frequently reported TEAEs in the 72 h after PLX-R18 administration were injection site reactions, such as pain, erythema, bruising and induration. These local events were transient and of mild to moderate severity. Additionally, some systemic events were reported in the 72 h after PLX-R18 therapy and may be related to hypersensitivity. These include tachycardia (1 patient, Cohort 3), hypotension (2 patients, Cohort 3), chills (2 patients, Cohort 3), peripheral edema (1 patient in Cohort 2, 1 patient in Cohort 3), pyrexia (4 patient, Cohort 3), and confusional state (1 patient, Cohort 3). The most common TEAEs are presented in Table 3.

**Table 3:** Most common TEAEs (reported in at least 20% of patients) by MedDRA preferred term (PT) seriousness.

Preferred Term	PLX-R18 1 M cells/kg			PLX-R18 2 M cells/kg			PLX-R18 4 M cells/kg			Overall		
	(Cohort 1) *N* = 3			(Cohort 2) *N* = 6			(Cohort 3) *N* = 12			*N* = 21		
	*n* (%)	Serious	Non-Serious	*n* (%)	Serious	Non-Serious	*n* (%)	Serious	Non-Serious	*n* (%)	Serious	Non-Serious
Injection site pain	3 (100.0)	0	3	3 (50.0)	0	3	12 (100.0)	0	12	18 (85.7)	0	18
Cough	2 (66.7)	0	2	1 (16.7)	0	1	5 (41.7)	0	5	8 (38.1)	0	8
Oedema peripheral	1 (33.3)	0	1	2 (33.3)	0	2	4 (33.3)	0	4	7 (33.3)	0	7
Constipation	1 (33.3)	0	1	1 (16.7)	0	1	4 (33.3)	0	4	6 (28.6)	0	6
Nausea	1 (33.3)	0	1	1 (16.7)	0	1	4 (33.3)	0	4	6 (28.6)	0	6
Pyrexia	1 (33.3)	0	1	0	0	0	5 (41.7)	1	4	6 (28.6)	1	5
Vomiting	2 (66.7)	0	2	1 (16.7)	0	1	3 (25.0)	0	3	6 (28.6)	0	6
Diarrhoea	2 (66.7)	0	2	0	0	0	3 (25.0)	0	3	5 (23.8)	0	5
Headache	2 (66.7)	0	2	0	0	0	3 (25.0)	0	3	5 (23.8)	0	5

Most frequent TEAEs defined as reported in ≥ 20% of the patients overall (i.e., reported in 5 or more patients).

No clinically meaningful changes were observed in vital signs, ECG and blood counts, liver and kidney function, or coagulation laboratory results. A transient and reversible elevation in inflammatory markers, high-sensitivity C-reactive protein (hsCRP) and complement factor 5a (C5a), were observed on the days after PLX-R18 administration, returned to normal levels thereafter, and were not associated with clinical manifestations. Key vital signs and laboratory parameters are presented in Fig. S8. As PLX-R18 is an allogeneic cell-based therapy, anti-HLA antibodies were assessed prior to treatment and during follow-up. No specific pattern indicating allo-sensitization could be detected (see Fig. S9).

### Exploratory efficacy

#### Recovery of blood counts

**Fig. 2:**
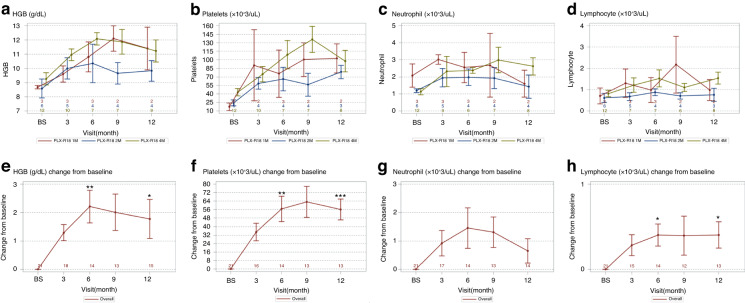
Mean values and change from baseline for hemoglobin, neutrophils, platelets, and lymphocytes. The top panel (**a**–**d**) presents mean values measured in peripheral blood for hemoglobin (HGB, **a**), Platelets (**b**), Neutrophils (**c**) and Lymphocytes (**d**) from baseline (prior to study treatment with PLX-R18) and over the duration of follow up, by the administered dose of PLX-R18: 1 million cells/kg (red), 2 million cells/kg (blue) and 4 million cells/kg (green). The number of available results is presented in a corresponding color above the x-axis. The bottom panel (**e**–**h**) presents change from baseline in mean values measured in peripheral blood for hemoglobin (HGB, **e**), Platelets (**f**), Neutrophils (**g**) and Lymphocytes (**h**) from baseline (prior to study treatment with PLX-R18) and over the duration of follow up. Data are presented for all treated patients as an overall cohort. The number of available observations is presented above the x-axis. *p < 0.05, **p < 0.001, ***p < 0.0001.

To better elucidate a potential treatment response, we first assessed response by exploring mean values over time and change from baseline in PLT, ANC, Hb, and lymphocyte levels both for the three cohorts separately and for all doses grouped as a pooled population (Fig. 2). In the pooled population, an increase from baseline is observed at all timepoints, with a peak at month 6 for Hb (*p* = 0.002) and lymphocytes (*p* = 0.008) and at month 9 for PLT (*p* < 0.001). The increase was maintained until month 12 for Hb (*p* = 0.02), lymphocytes (*p* = 0.02) and PLT (*p* < 0.0001). A non-significant increase was observed at month 6 for ANC (*p* = 0.063), returning to baseline values at month 12 (*p* = 0.15).

#### Hematologic Response (HR)

An attempt was made to assess HR using a modification of the 2018 version of the MDS-IWG (Myelodysplastic Syndrome International Working Group, see Table S10). At baseline, all patients had PLT below the responder threshold, 8 patients were above the responder threshold for Hb, and 14 patients were above the responder threshold for ANC. HR was defined as counts above the threshold in 2 or more cell lines at month 6. For patients who entered the study with only 1 cell line below the threshold, HR was defined as being above the threshold for this cell line and maintaining other counts above the threshold. The proportion of patients with a positive HR increased from 0% at baseline to 65% at month 6 (see Table S11). HR was also assessed in sub-groups of interest: HR was 86% for platelet transfusion independent patients vs. 50% for transfusion dependent patients, and 71% for RBC transfusion independent patients vs. 60% for transfusion dependent. Age did not impact HR. However, female patients had a reduced HR (50%) compared to males (73%). Additionally, a shorter duration from HCT ( < 240 days) was associated with increased HR, 80% vs. 43%. HR was similar for patients who did and those who did not use HGF, regardless of the time of use (i.e., at baseline or at any time to day 180). Data are summarized in Table S12.

#### Transfusion Requirements

**Fig. 3:**
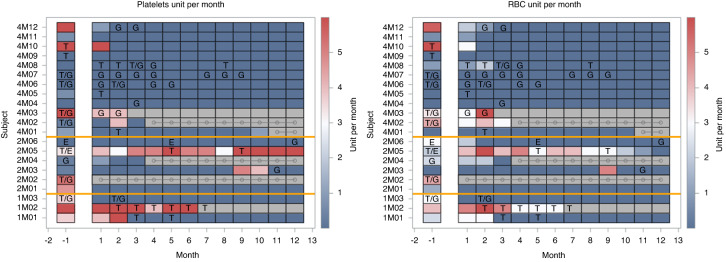
Platelet and RBC transfusion rate per month over time, patient level presentation. The color of a rectangle for a patient in a specific month indicates the number of units of platelets (left) or RBC (right) that the patient had received during that month. Blue indicates a small number of units or none, while red indicates more than six units. For presentation purposes, patients receiving more than six transfusion units per month, were fixed to six units. Grey rectangles indicate patients who discontinued follow up, horizontal grey lines indicate patients who died. Month “-1” presents a mean monthly value prior to study drug administration (the number of units for the 3 months preceding baseline were summed and divided by 3). From month 1 onward, the value represents the number of units for a specific month for that patient. The letters represent hematopoietic growth factor use: T = TPO receptor agonists, G = colony stimulating factors (G-CSF, GM-CSF), E = recombinant erythropoietin.

The need for transfusions was analyzed at baseline (a monthly mean of transfused units in the three months prior to the first PLX-R18 administration), and at all visits from first PLX-R18 administration. Patient-level data, including use of HGF, are presented in a heat map (Fig. 3) and in Fig. S13. The mean number of blood product transfusions per month in the overall population is presented in Table 4. For platelet transfusions, the monthly mean dropped from 5.09 units at baseline to 1.0 units by month 6 (*p*-value = 0.062) and 0.55 by Month 12 (*p*-value = 0.045). For RBC transfusions, the monthly mean dropped from 2.91 units at baseline to 0.58 units by month 6 (*p*-value = 0.0089) and 0 by Month 12 (*p*-value = 0.0005). While 11 of the 21 patients received HGF prior to study entry, HGF use was reduced at month 3 and was sporadic thereafter. There is no clear relationship between HGF use and the reduction in transfusion requirement.

**Table 4:** Mean platelet and RBC transfusions per month.

Month	Platelets				RBC			
	*n*	mean	std	*p*-value	*n*	mean	std	*p*-value
**Baseline**	15	5.1	7.1		15	2.9	1.8	
**3**	14	1	2.4	0.068	14	1	1.8	0.011
**4**	12	0.8	1.8	0.049	12	0.7	1.6	0.016
**5**	12	1.1	2.8	0.077	12	0.5	1.2	0.005
**6**	12	1	2.4	0.062	12	0.6	1.4	0.009
**7**	12	0.8	1.8	0.049	12	0.7	1.6	0.013
**8**	12	0.3	0.9	0.031	12	0.3	0.9	0.003
**9**	12	1.2	2.9	0.091	12	0.7	1.6	0.039
**10**	12	1.2	2.7	0.089	12	0.2	0.6	0.001
**11**	11	1.1	3.6	0.087	12	0.1	0.3	0.0008
**12**	11	0.55	1.8	0.045	12	0.0	0	0.0005

*RBC* Red Blood Cells, *Std* Standard deviation.

The *p*-value was calculated according to the t-test method with an equal variance assumption that was verified by an *F-*test.

#### Quality of life

The small cohort size does not support unambiguous conclusions as to SF-36 results (Tables S14–S16).

## Discussion

PGF is an unmet medical need, associated with increased mortality post-HCT [4, 19, 24, 25]. A lack of standardized definitions of PGF, number and thresholds of cytopenia required, response and the timing of its assessment make the comparison of our results to other cohorts challenging. Recognizing the substantial heterogeneity in studied PGF cohorts, and its impact on describing predictors of clinical outcome, Muskens et al. [25] explored survival in 566 PGF patients in a meta-analysis. Overall survival, reported from 1 to 5 years, was 53% and was not impacted by treatment regimen, cytopenia threshold, underlying disease, timing of PGF definition or its duration, year of study start or follow-up duration. Overall survival was higher in secondary vs. primary PGF, 61% vs. 40%, respectively. While our study did not differentiate between primary and secondary PGF, the observed 81% 12-month survival is comparable with these rates.

The current standard of care for PGF includes repeated blood product transfusions and HGF. An additional alternative is CD34+ cell boost, which shows encouraging results, but is limited by donor availability, the need to mobilize, extract, and isolate donor cells, and some risk of GvHD associated with the presence of CD3+ cells. In a meta-analysis of 7 retrospective studies reporting CD34+ cell boost for PGF [8], the overall response rate (ORR) was 80% (72-100% for individual studies) and the 12-month survival rate was 80%. While a direct comparison is not possible due to non-uniform definitions of cytopenia and response across studies, our study shows a 65% HR and 81% 12-month survival, with the advantages of using an off-the-shelf product with no risk of GvHD or additional donor involvement. The median time from HCT to treatment was shorter for CD34+ cell boost compared to this study (135 days vs. 238 days), which indicates a lower likelihood of spontaneous recovery in our study.

Outcomes from studies using TPO RA range from 45% to 75% ORR with a 60% 12-month overall survival [9, 26]. Allo-MSCs have been previously used in small cohorts of patients with cytopenia post-HCT. IV infusion of third-party BM MSCs, or BM-MSCs sourced from the original allo-HCT donors, were associated with a 30-100% response rate [13–15]. The variable response rate may be linked to donor identity, with third-party donors showing better response [14, 15]. PLX-R18 cells are sourced from young, healthy third-party donors offering a potential advantage. Placental-derived MSCs are considered comparable, and sometimes superior, to MSCs from other sources [27].

Time to peripheral blood count recovery post-HCT depends on many factors, and ranges from 2 to 24 months [9, 19]; a large case series reported a median time to recovery of 230 days [28]. Treatment with PLX-R18, in all tested doses, supported peripheral blood count recovery post-HCT with no clear dose response. Following two administrations during the first week, the increase in peripheral counts continued for months. The longest impact was observed for PLT, peaking at month 9, noteworthy as the most common cytopenia at baseline was thrombocytopenia. A clinically meaningful reduction in transfusion requirement was maintained for 12 months. The increase in overall lymphocyte count, which needs to be better explored in future studies, may support immune reconstitution post-HCT.

Our study demonstrated an acceptable safety profile of PLX-R18 in all tested doses. Injection site reactions were not serious and resolved spontaneously. The observed serious TEAEs of febrile neutropenia (3 events, 1 considered related) and malignancy recurrence (1 event) are within expected rates in this patient population [29–31]. No discernable pattern of anti-HLA antibody development was observed, supporting the proposed immune-evasive nature of PLX-R18, potentially associated with the placental cell source.

Study limitations include a small sample size, especially for lower doses; lack of a control arm, limiting the ability to rule out spontaneous recovery as an explanation for the results; and a heterogeneous population on several parameters, such as onset PGF post-HCT, underlying disease, HCT cell source, and baseline blood counts. These limitations make the interpretation of the results challenging. However, both HR and survival rates observed in our study mirror rates reported in the literature [19, 25].

The IM route of administration may also be considered a limitation due to the discomfort and potential for bleeding in thrombocytopenic patients. As is observed for MSCs in the post-HCT setting [19], when PLX-R18 was administered IV in pre-clinical studies, the cells migrated mostly to the lungs and were cleared more quickly compared to IM administration [21]. Considering the proposed paracrine mechanism of action, longer persistence justified the IM administration. The theoretical concern of bleeding did not materialize in our study.

The results of our study are encouraging and indicate a potential role for PLX-R18 in accelerating hematopoietic recovery post-HCT. Additional studies are warranted to optimize the efficacy of PLX-R18 in the treatment of PGF and to expand the potential to other BM failure disorders and cytopenia associated with certain treatment modalities such as CAR-T therapy.

## Supplementary information

Supplemental material document

## Data Availability

The data that support the findings of this study are available from Pluri-Biotech, Ltd. but restrictions apply to the availability of these data, which were used under license for the current study, and so are not publicly available. Data are, however, available from the authors upon reasonable request and with permission of Pluri-Biotech, Ltd.
