# Correction: Placental expanded mesenchymal-like cells (PLX-R18) for poor graft function after hematopoietic cell transplantation: A phase I study

**DOI:** 10.1038/s41409-024-02469-y

**Published:** 2024-12-03

**Authors:** Joseph P. McGuirk, Leland Metheny, Luis Pineiro, Mark Litzow, Scott D. Rowley, Batia Avni, Roni Tamari, Hillard M. Lazarus, Jacob M. Rowe, Michal Sheleg, Daniel Rothenstein, Nitsan Halevy, Tsila Zuckerman

**Affiliations:** 1 Division of Hematologic Malignancies & Cellular Therapeutics, University of Kansas Medical Center, Kansas City, KS USA; 2 Case Western Reserve University, Cleveland, OH USA; 3 University Hospitals Seidman Cancer Center, Cleveland, OH USA; 4 Apheresis and Marrow Processing Laboratories, Baylor University Medical Center, Dallas, TX USA; 5 Division of Hematology, Mayo Clinic, Rochester, MN USA; 6 Stem Cell Transplantation and Cellular Therapy Program, John Theurer Cancer Center, Hackensack, NJ USA; 7 Hadassah University Medical Center, Jerusalem, Israel; 8 Memorial Sloan Kettering Cancer Center, New York, NY USA; 9 Case Western Reserve University School of Medicine, Cleveland, OH USA; 10 Department of Hematology, Shaare Zedek Medical Center, Jerusalem, Israel; 11 The Ruth and Bruce Rappaport Faculty of Medicine, Technion, Haifa, Israel; 12 Department of Hematology and Bone Marrow Transplantation, Rambam Health Care Campus, Haifa, Israel; 13 Pluri-Biotech Ltd., Haifa, Israel

**Keywords:** Drug development, Haematopoietic stem cells, Haematological diseases, Therapeutics, Bone marrow transplantation

Correction to: *Bone Marrow Transplantation* 10.1038/s41409-023-02068-3, published online 08 August 2023

Following the publication of this article, the authors would like to add an additional reference as below to add context on the original discovery of PLX-R18's proposed therapeutic capabilities:

16. Gaberman E, Pinzur L, Levdansky L, Tsirlin M, Nete~ N, Aberman Z, et al. Mitigation of Lethal Radiation Syndrome in Mice by Intramuscular Injection of 3D Cultured Adherent Human Placental Cells. PLoS One. 2013;8(6):e66549

The remaining references have been relabelled accordingly.

The authors also noted the omission of ‘et al’ in the below reference:

17. Pinzur L, Akyuez L, Levdansky L, Blumenfeld M, Volinsky E, Aberman Z, et al. Rescue from Lethal Acute Radiation Syndrome (ARS) with Severe Weight Loss by Secretome of Intramuscularly Injected Human Placental Stromal Cells. Journal of Cachexia Sarcopania and Muscle. 2018;9:1079-1092

The original article has been corrected.

