# Supplementary material for: Placental expanded mesenchymal-like cells (PLX-R18) for poor graft function after hematopoietic cell transplantation: A phase I study
Source: Bone Marrow Transplant. 2023 Aug 8;58(11):1189–96. doi: 10.1038/s41409-023-02068-3 (PMC10622312; doi:10.1038/s41409-023-02068-3)
Supplement: Supplementary file 1 — Supplemental material document [file 41409_2023_2068_MOESM1_ESM.docx]

**suplamentary material**

S1: Selected secreted factors, PLX-R18

S2: Inclusion/exclusion criteria

S3: Details of the PLX-R18 manufacturing process

S4: Schedule of assessments

S5: Prespecified exploratory efficacy outcomes

S6: Summary of screen failures

S7: Brief narratives of TEAEs with a fatal outcome

S8: Box plots of vital signs and laboratory parameters

S9: Patient-level display of anti-HLA antibodies

S10: Modified IWG criteria used to define hematologic response

S11: Proportion of patients with a positive hematologic response based on modified IWG criteria

S12: Sub-group analysis of positive hematologic response at month 6, by various parameters

S13: Patient level presentation of blood counts, use of blood product transfusions and hematopoietic growth factors

S14: SF-36V2 Quality of life scores over time by treatment cohort

S15: SF-36V2 Quality of life scores over time for the overall population

S16: SF-36V2 Quality of life analysis by physical and mental component summary scales

S17: List of ethics committees/institutional review boards

**Table S1: Selected secreted factors, PLX-R18**

| **Protein** | **number of tested batches** | **MEAN**  **pg/mL** | **Std**  **pg/mL** | **CV** | **min**  **pg/mL** | **max**  **pg/mL** | **Method of measurment** |
| --- | --- | --- | --- | --- | --- | --- | --- |
| **IL-6** | 4 | 39726.2 | 10166.9 | 25.6 | 30467.8 | 53462.4 | **Luminex** |
| **G-CSF** | 4 | 6903.4 | 3277.8 | 47.5 | 2606.8 | 10193.7 | **Luminex** |
| **IL-8** | 4 | 6760.1 | 606.2 | 9.0 | 6044.0 | 7483.3 | **Luminex** |
| **M-CSF** | 3 | 2470.8 | 214.0 | 8.7 | 2270.9 | 2696.5 | **ELISA** |
| **GM-CSF** | 4 | 1723.1 | 1741.3 | 101.1 | 322.8 | 4017.2 | **Luminex** |
| **MCP-1** | 4 | 1515.0 | 1286.9 | 84.9 | 267.0 | 3267.4 | **Luminex** |
| **IL-11** | 3 | 349.6 | 89.1 | 25.5 | 269.6 | 445.6 | **ELISA** |
| **SCF** | 3 | 131.2 | 53.7 | 41.0 | 78.0 | 185.5 | **ELISA** |
| **IL-3** | 4 | 44.7 | 6.6 | 14.8 | 38.0 | 52.3 | **Luminex** |
| **IL-2** | 4 | 15.0 | 2.2 | 14.4 | 13.2 | 17.9 | **Luminex** |
| **IL-7** | 4 | 11.0 | 2.2 | 19.9 | 9.0 | 14.1 | **Luminex** |
| **IL-5** | 4 | 9.1 | 0.8 | 9.3 | 8.1 | 9.9 | **Luminex** |
| **IL-4** | 4 | 3.1 | 0.5 | 14.3 | 2.6 | 3.7 | **Luminex** |

CV – Coefficient of Variation, defined as CV=(STD/Mean)*100

**List S2: Inclusion/Exclusion criteria**

**Inclusion Criteria**

Patients were eligible for inclusion in the study if they met all of the following criteria:

1. Age ≥ 18 years
2. At least three months after HCT, either autologous or allogeneic (of any source, with any preparatory regimen, for any indication), prior to study treatment
3. Sustained platelet count ≤ 50,000/µL and/or sustained Hb ≤ 8 g/dL and/or sustained ANC ≤ 1000/mm^3^ attributed to graft failure as a major contributor, as evident by relative hypocellular BM

* Cytopenia was to be confirmed by at least two consecutive blood counts, at least one of them within 28 days prior to treatment (higher transient levels following occasional blood product transfusions were allowed)

1. Stable donor cell chimerism in at least three consecutive tests prior to treatment (the most recent test had to be within 28 days prior to treatment)
2. If the patient had allogeneic HCT for a malignant disease, the patient had to have complete donor chimerism (complete donor chimerism had to be determined by the Investigator per site’s standards)
3. General performance status 0-2 evaluated by the Eastern Cooperative Oncology Group (ECOG) scale
4. Signed written informed consent

**Exclusion Criteria**

Patients were to be excluded from the study if they met any of the following criteria:

1. Evidence of developing malignancy since the HCT, or any evidence of malignancy at the time of screening
2. Active infection requiring systemic treatment at the time of screening (if infection resolved but antibiotic coverage continued, the patient could be included)
3. GvHD Grade III or IV, or severe chronic GvHD at the time of screening
4. Patient had received prophylactic treatment with donor lymphocyte infusion (DLI) within six months prior to treatment, or any other cell therapy within three months prior to treatment
5. History of malignancy (other than the disease that required the HCT) within two years prior to screening (except for skin basal cell carcinoma or squamous cell carcinoma lesions that were fully resected with no need for further treatment, and not located at the injection site)
6. History of significant transfusion reaction, including transfusion‑related acute lung injury (pulmonary edema), shock, severe disturbances of liver function tests, renal dysfunction, or hemolytic anemia (as part of the transfusion reaction)
7. Known allergies to any of the following: dimethyl sulfoxide (DMSO), human serum albumin, bovine serum albumin, gentamicin, or antihistamine
8. History of allergic/hypersensitivity reaction to any substance having required hospitalization and/or treatment with intra-venous steroids/epinephrine, or in the opinion of the Investigator the patient was at high risk of developing severe allergic/hypersensitivity reactions (does not apply to transfusion reactions)
9. A known history of allergic/hypersensitivity reactions to three or more allergens
10. History of uncontrolled asthma (Global Initiative for Asthma Grade III‑IV)
11. History of severe atopic disease (including but not limited to chronic urticaria, allergic reaction with respiratory symptoms requiring systemic steroids)
12. Medical history of human immunodeficiency virus or syphilis infection
13. Known active hepatitis B or hepatitis C infection at the time of screening
14. A pregnant or lactating woman or a woman who planned to become pregnant during the study. In addition, any woman of childbearing potential (not sterile or postmenopausal), who was unwilling to adhere to the use of a highly effective contraception method for the duration of the study:
    1. Oral/intravaginal/transdermal combined estrogen and progestogen containing hormonal contraception for at least three months prior to screening
    2. Oral/injectable/implantable progestogen-only hormonal contraception for at least three months prior to screening
    3. An intrauterine device (IUD) or intrauterine hormone-releasing system (IUS)
15. Patients on renal replacement therapy or with estimated glomerular filtration rate (eGFR) < 15 mL/min/1.73m^2^ (based on Modification of Diet in Renal Disease [MDRD] equation)
16. Serum‑glutamic pyruvic transaminase (alanine aminotransferase), serum‑oxaloacetic pyruvic transaminase (aspartate aminotransferase) > 2.5 x upper limit of normal range
17. International normalized ratio (INR) > 2 or patients who were on oral anticoagulant therapy with INR > 2 unless anticoagulation treatment could be safely interrupted/discontinued around each IP treatment upon primary care physician and/or Investigator’s discretion
18. Severe or uncontrolled/unstable cardiac, pulmonary, or renal disease, including myocardial infarction or cerebrovascular accident within three months prior to treatment
19. History of solid organ transplantation
20. Signs and symptoms of active central nervous system disease
21. Life expectancy of less than six months as assessed by the Investigator
22. Patient had participated in a clinical interventional study and received the last treatment within 30 days prior to screening
23. In the opinion of the Investigator, the patient was unsuitable for participating in the study

**Supplement information S3: Details of the PLX-R18 manufacturing process**

The manufacturing process consists of two stages, previously reported in Papait A et al., 2020 (Reference 17 of the main text). The cells are initially digested from the placenta and expanded in 2-dimensional (2D) for several passages, after which they concentrated and cryopreserved in vials containing an Intermediate Cell Stock (ICS). In the second stage, ICS is thawed and cultured in 2D for additional passages until the culture reaches 60–90% confluency and then transferred to bioreactors for a final culture in an automatically controlled 3D-expansion on carriers to produce the final PLX-R18 product. Generally, the cells do not exceed 25 population doublings; batches used in this study were in the range of 19-25 population doublings.

Until mid-2018 PLX-R18 was provided in a final formulation containing 10% DMSO (v/v) and 5% human serum albumin (HAS) (w/v) in Plasma-Lyte (“old formulation”). After mid-2018, PLX-R18 was provided as a frozen cell dispersion in 6 mL vials, at a concentration of either 10 million cells/mL or 20 million cells/mL, in a solution containing 4% DMSO (v/v) and 5% HSA (w/v) in Plasma-Lyte (“new formulation”).

**Table S4: Schedule of Assessments**

| **Study procedure** | **Screening** | **First treatment** | | **Second treatment** | | **Follow-up** | | | | | | **Termination / Early discontinuation** | **Unscheduled** |
| --- | --- | --- | --- | --- | --- | --- | --- | --- | --- | --- | --- | --- | --- |
| **Visit number** |  | 1 | 2 | 3 | 4 | 5 | 6 | 7 | 8 | 9 | 10 | 11 |  |
| **Week in study** | -4 to 0 |  |  | 1 |  | 2 | 4 | 8 | 12 | 24 | 36 | 52 |  |
| **Day in study** | -28 to 0 | 0 | 1  (24±3h) | 7 | 8+1 (24±3h from second treatment)* | 14+1 | 28±2 | 56±3 | 84±7 | 168±14 | 252±14 | 364±14 (1 year) |  |
| Obtain written informed consent | X |  |  |  |  |  |  |  |  |  |  |  |  |
| Assign screening number | X |  |  |  |  |  |  |  |  |  |  |  |  |
| Inclusion/Exclusion criteria | X |  |  |  |  |  |  |  |  |  |  |  |  |
| Confirm eligibility ^a^ |  | X |  |  |  |  |  |  |  |  |  |  |  |
| Sponsor screening assessment confirmation | X |  |  |  |  |  |  |  |  |  |  |  |  |
| Investigator orders Study IP from the Sponsor | X |  |  |  |  |  |  |  |  |  |  |  |  |
| Demographic and medical history | X |  |  |  |  |  |  |  |  |  |  |  |  |
| Treatment history and ongoing medications | X |  |  |  |  |  |  |  |  |  |  |  |  |
| Allergy history | X |  |  |  |  |  |  |  |  |  |  |  |  |
| Body weight and height | X ^c^ |  |  |  |  |  |  |  |  |  |  | X ^c^ |  |
| BM aspiration and biopsy (central lab) ^d^ | X |  |  |  |  |  |  |  | X |  |  |  |  |
| Chimerism assessment ^e^ | X ^e^ |  |  |  |  |  | X | X | X ^e^ | X | X | X |  |
| ECG ^f^ | X | X ^f^ |  | X ^f^ |  | X |  |  |  |  |  | X | X (if necessary) |
| Pre-treatment with antihistamine |  | X |  | X |  |  |  |  |  |  |  |  |  |
| Treatment with PLX‑R18 ^g^ |  | X |  | X ^h^ |  |  |  |  |  |  |  |  |  |
| Vital signs ^i^ | X | X | X | X | X | X | X | X | X | X | X | X | X |
| Blood chemistry and CBC with differential ^j, k, l^ | X | X | X | X | X | X | X | X | X | X | X | X | X |
| Coagulation profile ^m^ | X | X |  | X |  |  |  |  |  |  |  |  |  |
| Blood sample for cytokines ^n^ |  | X | X | X | X | X |  |  |  |  |  |  |  |
| Anti‑HLA antibodies ^o^ |  | X |  |  |  |  | X |  |  | X |  | X |  |
| Pregnancy test ^p^ | X | X |  | X |  |  |  |  |  |  |  | X |  |
| EBV and CMV viral load (PCR) | X |  |  |  |  | X | X | X | X | X | X | X |  |
| Urinalysis ^q^ | X | X |  | X |  |  |  |  |  |  |  | X |  |
| Physical examination ^r^ | X |  | X |  | X |  |  |  |  |  |  | X | X (if necessary) |
| GvHD assessment |  | X |  |  |  |  | X | X | X | X | X | X |  |
| Record Lung Function Test results (if done as part of standard care) ^s^ | X | X | X | X | X | X | X | X | X | X | X | X |  |
| Record AEs ^t^ |  | X | X | X | X | X | X | X | X | X | X | X | X |
| Record all concomitant medication | X | X | X | X | X | X | X | X | X | X | X | X | X |
| Documentation of blood transfusion products | X | X | X | X | X | X | X | X | X | X | X | X | X |
| QoL assessment (via SF36v2 questionnaire) |  | X |  |  |  |  | X |  | X | X |  | X |  |
| Flow cytometry (sub-population FACS) ^u^ | X | X |  |  |  | X |  |  | X | X |  | X |  |
| Blood sample for C5a ^v^ |  | X | X | X | X | X |  |  |  |  |  |  |  |
| Blood samples for histamine and tryptase level ^w^ |  | X |  | X |  |  |  |  |  |  |  |  |  |

AEs=adverse events; BM=bone marrow; CBC=complete blood count; CMV=cytomegalovirus; EBV=Epstein-Barr virus; ECG=electrocardiogram; FACS= fluorescence-activated cell sorting; GvHD=graft versus host disease; HLA=human leukocyte antigen; IP=investigational product; PCR= polymerase chain reaction; QoL=quality of life

* Visit 4 was to begin 24 ± 3h following investigational product (IP) administration on Visit 3.

^a^ For confirmation of eligibility, blood counts from screening had to be reviewed.

^b^ IP ordering: was to be performed after assessment of study eligibility criteria at least six days prior to treatment and following the Sponsor’s screening assessment confirmation. All available results of complete blood count (CBC) performed during the screening period were to be reviewed prior to IP ordering.

^c^ Height and weight were to be measured at screening; weight was also to be measured at Visit 11.

^d^ It was recommended to perform bone marrow (BM) aspiration and biopsy within the screening period, up to 28 days prior to study treatment. However, if not possible, results from a BM aspiration/biopsy done up to 60 days before planned first treatment could be used. If possible, site was to send to the central laboratory, as well as the local lab available samples/blocks for confirmatory testing.

^e^ Chimerism: at Visit 8 a BM sample was to be used; at all other visits a blood sample was to be used.

^f^ ECG had to be performed prior to IP administration at Visits 1 and 3.

^g^ Each individual dose was to be calculated for each patient based on cohort assignment and the patient’s body weight at screening. PLX-R18 was to be injected in multiple intramuscular (IM) injections: half of the injections to the to the gluteus medius on one side, and half of the injections to the thigh muscles on the contralateral side, until reaching the required total dose.

^h^ The second IP treatment was not to be administered to patients who, following first administration, develop any systemic allergic/hypersensitivity reaction (any Common Terminology Criteria for Adverse Events version 4 [CTCAE v4] grade), any severe (CTCAE v4 Grade 3-5) or serious local allergic/hypersensitivity reaction, or any allergic/hypersensitivity reaction that required treatment with intra-venous or oral steroids or with epinephrine, or to patients for whom, in the opinion of the Investigator, the risk of developing such allergic/hypersensitivity reactions had increased since screening. The second IP treatment could be administered to patients with non-serious and non-severe (CTCAE v4 Grade 1-2) local allergic/hypersensitivity reactions following the first administration. In case a stopping rule suspended the second administration of PLX-R18 to a patient, the administration of this second dose at a later time was to be decided with the Sponsor on a case-by-case basis.

^i^ Vital signs: blood pressure, heart rate, respiratory rate, temperature. At Visit 1 and Visit 3, vital signs were to be measured before IP administration, and 1 hour ± 15 minutes after IP administration.

^j^ Full biochemistry: glucose, blood urea nitrogen, creatinine, sodium, potassium, chloride, total protein, albumin, calcium, phosphorus, uric acid, total bilirubin, alkaline phosphatase, C‑reactive protein, aspartate aminotransferase, alanine aminotransferase.

^k^ CBC with differential: hemoglobin (Hb), hematocrit, red blood cell (RBC) count, RBC indices, platelet count, white blood cell (WBC) count and differential count absolute and percentages (polymorphonuclear leukocytes [neutrophils], lymphocytes, eosinophils, monocytes, basophils, atypical lymphocytes).

^l^ CBC was to be sent to both local and central laboratories prior to treatment at Visit1 and Visit 3. Local laboratory results of CBC were to be used to decide whether platelet transfusion could be considered before the IP administration.

^m^ Coagulation profile - activated partial thromboplastin time, partial thromboplastin time, and international normalized ratio.

^n^ In Visits 1 and 3 samples for cytokines were to be taken before PLX-R18 administration. Cytokines were to be measured using an approved immunology cytokine/chemokine multiplex assay. An additional blood sample for cytokine testing was to be taken two to four hours post IP administration.

^o^ HLA antibodies: on Visit 1 prior to treatment; the 12-month test was optional, and was to be done in case a new specificity was detected in previous anti-HLA antibody tests in comparison to baseline.

^p^ For women of childbearing potential a serum pregnancy test (β human chorionic gonadotropin) was to be performed at screening, and a urine pregnancy test was to be performed at all other time points.

^q^ Urinalysis: specific gravity, blood, bilirubin, glucose, ketones, protein, and pH.

^r^ Physical examination: included appearance, eyes, ears, nose, head, throat, neck, chest, lungs, heart, abdomen, extremities, skin, and musculoskeletal system.

^s^ Lung Function Test results were to be recorded only if done as part of standard care.

^t^ Adverse events (AEs): recorded from informed consent signature throughout the study until the last visit.

^u^ Fluorescence-activated cell sorting (FACS) analysis was optional and was only to be completed in a subset of patients at study sites with adequate capability. FACS analysis was to be done on BM at screening and at Visit 8, and on peripheral blood at screening and at Visits 1, 5, 9, and 11.

^v^ On Visits 1 and 3 C5a factor was to be tested before treatment.

^w^ On Visits 1 and 3 prior to treatment and two to four hours after treatment; blood samples were to be taken and stored for potential testing of tryptase and histamine levels in case of any suspected allergic reaction to PLX-R18.

**List S5: Prespecified Exploratory Efficacy Outcomes**

1. Change from baseline in platelet count
2. Change from baseline in Hb level
3. Change from baseline in ANC
4. Change in transfusion frequency as compared to the three months preceding study treatment
5. Shift from transfusion dependence to transfusion independence
6. Change in GvHD severity
7. Change in QoL via the SF-36v2 questionnaire
8. Change in levels of the serum immunological parameters anti-human leukocyte antigen (HLA) antibodies, C5a, and peripheral blood/BM sub-populations (by FACS)
9. Change in cytomegalovirus (CMV) or Epstein-Barr Virus (EBV) viral load
10. Change in serum cytokine levels
11. Change in BM biopsy examination

**Table S6: Summary of screen failures**

| **Subject name or identifier** | **Reason for screen failure** |
| --- | --- |
| 504-01 | ANC=0 and current infection |
| 503-01 | Inclusion #3, plt>50 |
| 504-03 | Malignancy within the past 5 years |
| 514-02 | Subject decided to withdraw and did not meet inclusion #3 |
| 504-05 | Exclusion# 23 & inclusion #3 |
| 514-05 | Sustained platelet count and Hgb during screening process |
| 504-07 | Exclusion #1 |
| 504-08 | Inclusion criteria #2, #4, and #5 |
| 602-01 | Pericarditis |
| 601-04 | Subject decided to withdraw |
| 602-02 | Exclusion criteria #16 |
| 512-02 | Exclusion criteria #1 |
| 502-01 | Exclusion criteria #1 |

**Supplement information S7: Brief Narratives of TEAEs with a Fatal Outcome**

Patient 514-01, Cohort 2: This was a 60-year-old female who underwent allogeneic HCT with BM cells on 30-January-2018 due to acute lymphoblastic leukemia diagnosed on 16-February-2017. The patient received myeloablative conditioning regimen with chemotherapy and total body irradiation. The patient was assigned to Cohort 2, and received the first treatment with PLX-R18 (152 million cells) on 11-July-2018, and the second treatment (150 million cells) on 18-July-2018. On 30-July-2018, 20 days after the first PLX-R18 treatment and 13 days after the second treatment, the patient developed “pneumonia” (serious, Grade 3) and was hospitalized on the next day. On the day of hospitalization the patient developed “sepsis” (serious, Grade 5). The patient was transfused and treated for sepsis and pneumonia from 31-July-2018 to 06-August-2018. On 02-August-2018, 23 days after the first and 16 days after the second PLX-R18 treatment the patient developed “acute kidney injury” (serious, Grade 3) and “respiratory failure” (serious, Grade 4). The patient was treated for acute kidney injury and sepsis, as well as for respiratory failure from 02-August-2018. The patient died on 06-August-2018, 27 days after the 1^st^ treatment with PLX-R18 and the cause of death was reported as “sepsis”. The Investigator assessed the event “sepsis” as serious due to hospitalization and death (Grade 5), not related to the study treatment, and related to pneumonia. The Sponsor also assessed the event “sepsis” as not related to PLX-R18 and related to patient’s underlying medical condition.

Patient 601-01, Cohort 2: This was a 54-year-old male who had allogeneic HCT with peripheral blood stem cells on 25-August-2015 and on 17-November-2017 due to myelofibrosis diagnosed on 19-September-1996. The patient received reduced-intensity conditioning regimens with chemotherapy. The patient was assigned to Cohort 2, and received the first treatment with PLX-R18 (130 million cells) on 24-June-2018 and the second treatment (130 million cells) on 01-July-2018. On 06-Sep-2018, 74 days after the 1^st^ and 67 days after the 2^nd^ PLX-R18 treatment, the patient fell and had a head and leg (right calf) injuries (serious, Grade 3) requiring stitches. The patient had a degloving wound with cutaneous flap and congested, purplish skin at the distal calf with exposed subcutaneous fat tissue. The flap was not closed due to the fear that the congestion would be exacerbated and would compromise the blood flow, and the patient was admitted to the General Surgery Department for further observation. The patient was discharged from the hospital on 08-Sep-2018 in hemodynamically and respiratory stable condition. On 29-September-2018, 98 days after the first and 91 days after the second PLX-R18 treatment, the patient experienced severe pain along his right calf with appearance of new erythema and swelling, and was hospitalized. The patient started to develop signs of septic shock with hypotension, acute respiratory distress, dyspnea and resistant hypoglycemia and fever, and he was ventilated. On 30-September-2018, the patient had an asystole. Unsuccessful resuscitation attempt was performed but the patient died on the same day. The cause of death was reported as septic shock. The Investigator assessed the event of “septic shock with acute respiratory distress” as serious (fatal and hospitalization), Grade 5, unrelated to the study treatment and related to possible infection due to leg injury. The Sponsor assessed the event of “septic shock with acute respiratory distress” as not related to PLX-R18 due to the patient’s underlying condition, including pancytopenia and multiple infections in the preceding months.

Patient 504-06, Cohort 3: This was a 63-year-old female who underwent allogeneic HCT with peripheral blood stem cells on 22-November-2017 due to Non-Hodgkin’s lymphoma which was diagnosed on 14-April-2016 and was treated with myeloablative conditioning regimen with chemotherapy. The patient was assigned to Cohort 3, and received the first treatment with PLX-R18 (340 million cells) on 19-June-2019 and the second treatment (340 million cells) on 26-June-2019. The patient was transfusion dependent and received the last RBC transfusion prior to PLX-R18 treatment on 12 and 18-June-2019. On 09-August-2019, 52 days from the first PLX-R18 treatment and 45 days from the second treatment, the patient experienced an upper respiratory infection (non-serious) and body petechia (non-serious) which resolved on 14-August-2019. She was treated for the upper respiratory infection until 25-September-2019, when the event was resolved.. On 16-October-2019, 120 days from the first PLX-R18 treatment and 113 days from the second treatment, the patient died (Grade 5), unwitnessed, at home. The reported cause of death was “sudden unexplained death”. No additional information about the patient’s clinical course in the days before the death was available. The Investigator assessed the event ‘sudden unexplained death’ as serious due to death, unlikely related to the study treatment and related to unknown cause. The Sponsor assessed the event ‘sudden unexplained death’ as not related to PLX-R18 due to lack of temporal relationship and the patient’s underlying condition.

Patient 503-04, Cohort 3: This was a 46-year-old male patient who underwent an allogenic HCT with umbilical cord blood cells on 19-April-2018 due to acute lymphoblastic leukemia which was diagnosed on 01-December-2017. The patient received myeloablative conditioning regimen with chemotherapy. The patient was assigned to Cohort 3, and received the first treatment with PLX-R18 (354 million cells) on 30-May-2019, and the second treatment (360 million cells) on 06-June-2019. On 21-March-2020, 297 days after the first and 290 days after the second PLX-R18 treatment the patient presented to the clinic with dizziness, weakness, abdominal pain radiating to his left shoulder, non-productive cough, and dyspnea with exertion. He was diagnosed with “acute lymphoblastic leukemia relapse” (serious, Grade 5). On 26-March-2020 the patient experienced excruciating abdominal and back pain, worsening of liver function, fever, and hypoglycemia. The patient also had minor but continuous epistaxis. On 27-March-2020, mild coagulopathy was noted, while on 30-March-2020 the patient continued to experience epistaxis. The patient received platelet transfusion on 27-March-2020, 28-March-2020, 30-March-2020, 01-April-2020 and 03-April-2020, and packed RBC transfusion on 29-March-2020. On 03-April-2020 the patient and his family decided to leave the hospital and moved to a hospice care facility, where the patient died on 04-April-2020 due to acute lymphoblastic leukemia relapse. The Investigator assessed the event “acute lymphoblastic leukemia relapse” as serious due to hospitalization and death (Grade 5), unrelated to the study treatment, and related to patient’s concurrent illness. Considering relapse is the most frequent cause of treatment failure after allogeneic HCT, accounting for death in 37–57% recipients after allogenic HCT (depending on the donor source used), and as the multiple chemotherapy treatment the patient received may have also contributed to the relapse, the Sponsor assessed the event as not related the PLX-R18.

**Figure S8: Box Plots of Vital Signs and Laboratory Assessments**

**
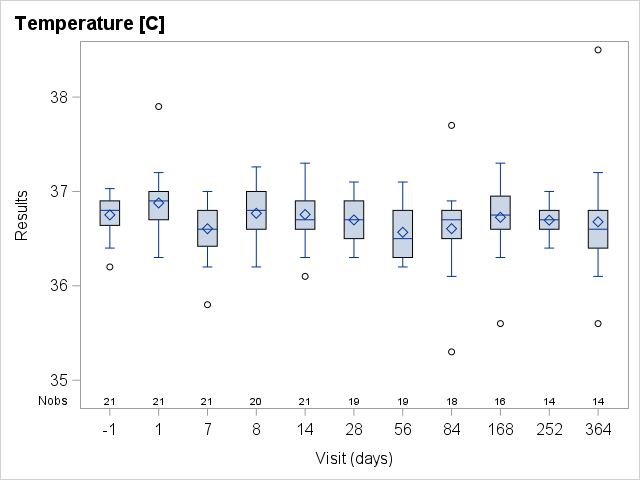
**

**
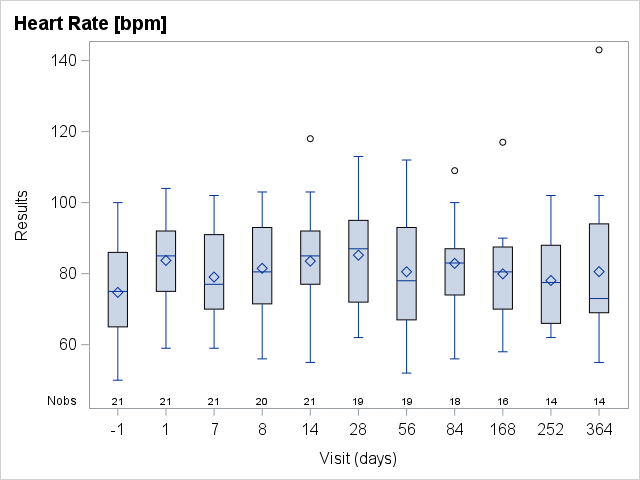

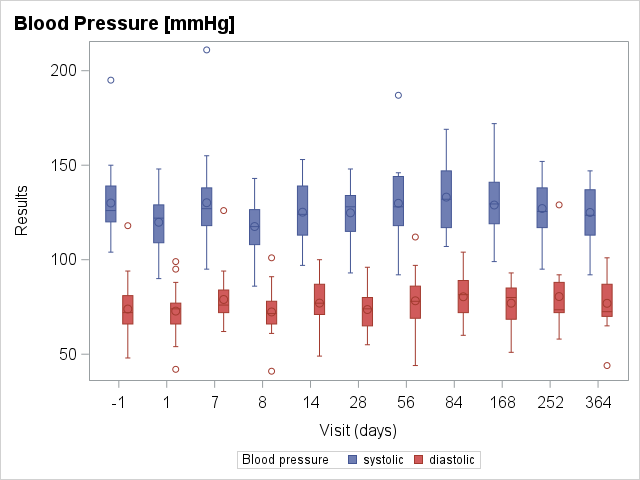
**

**
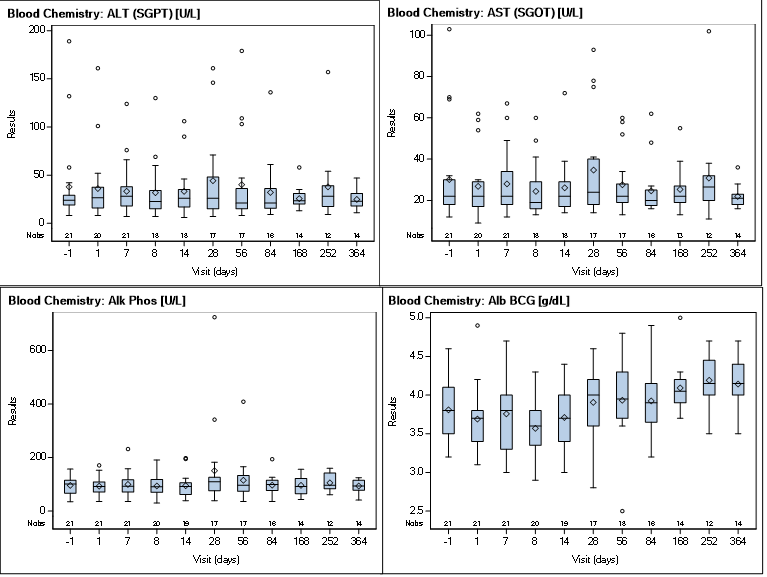
**

**
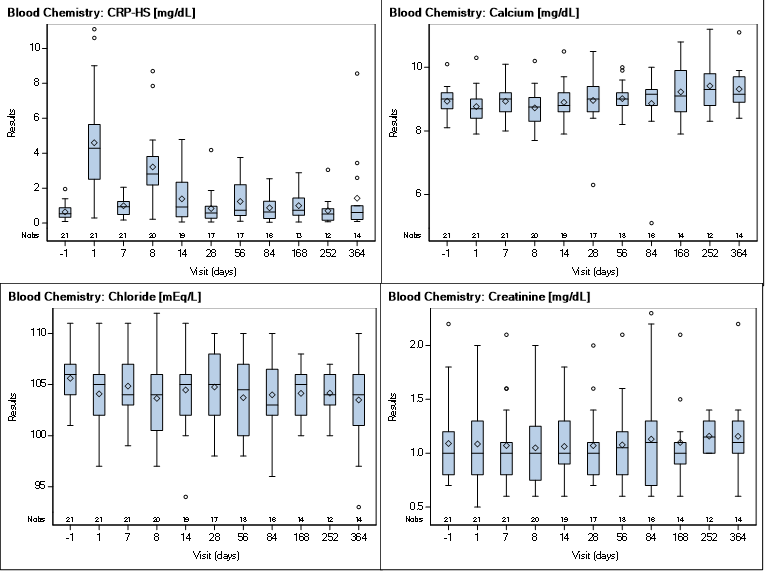
**

**
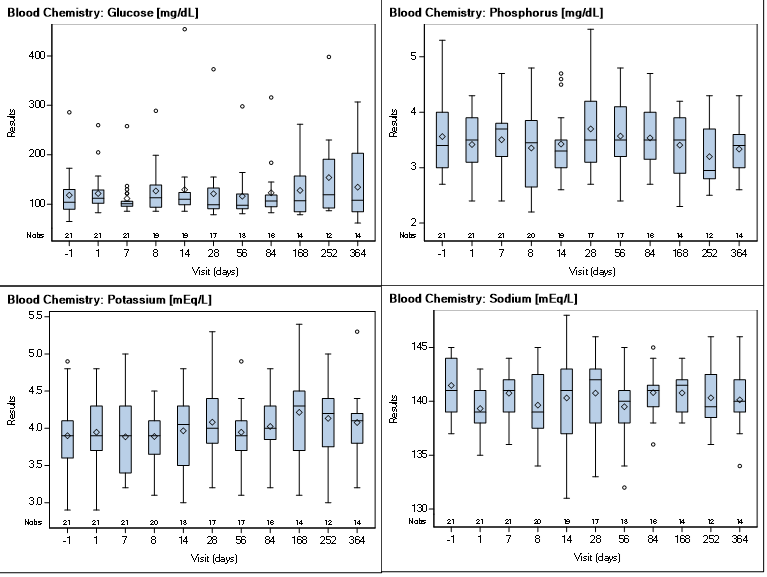
**

**
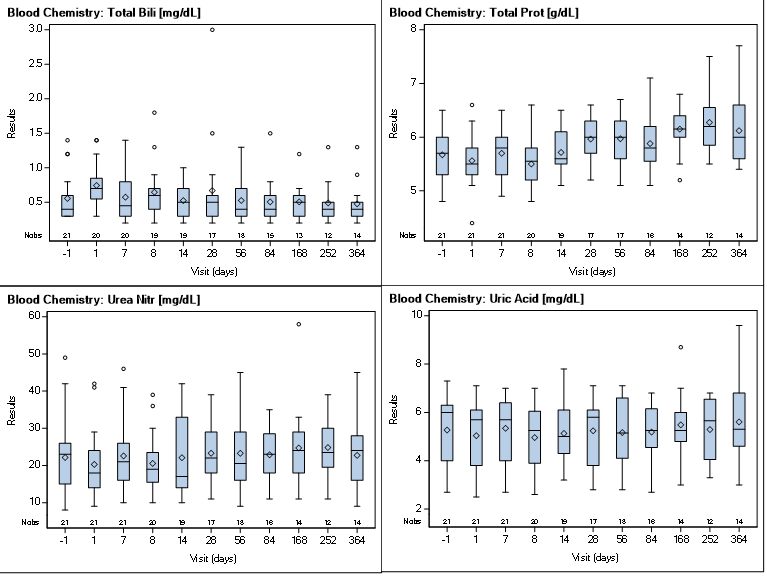
**

**
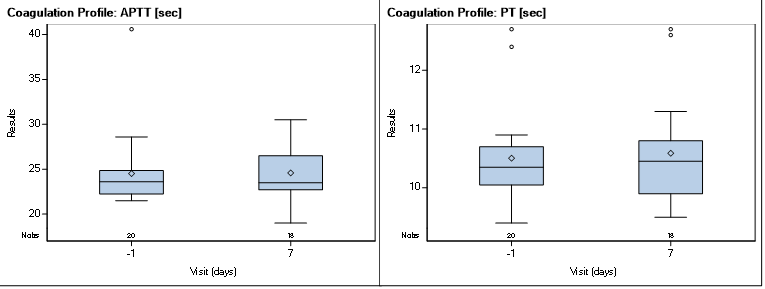
**

**
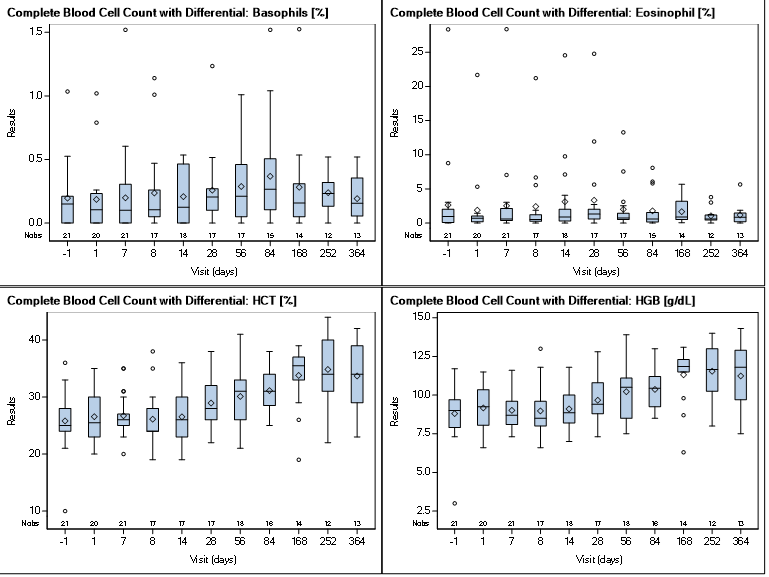
**

**
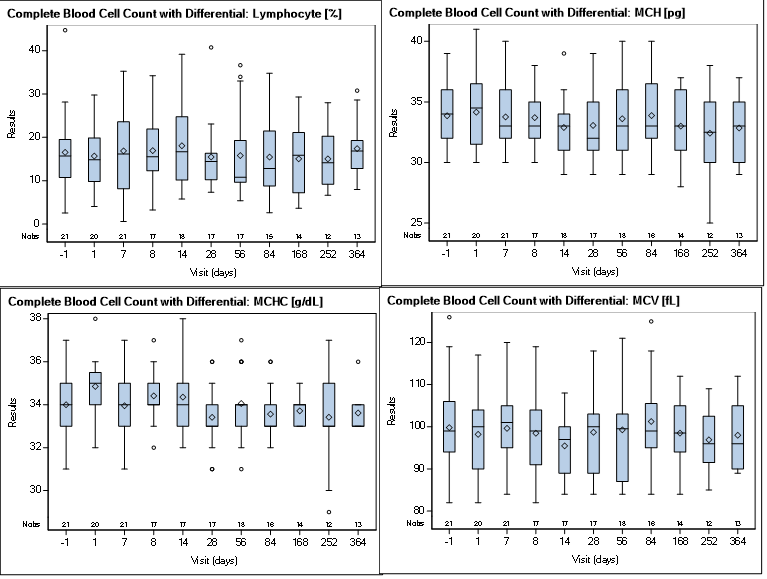
**

**
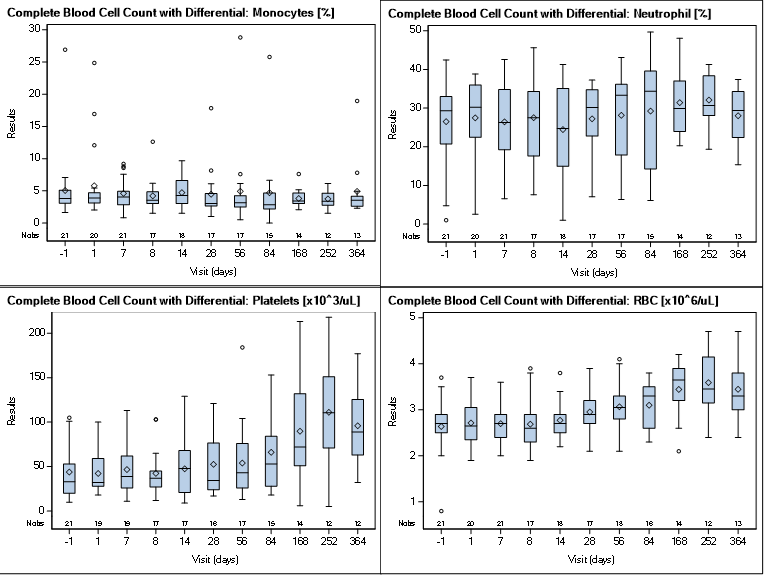
**

**
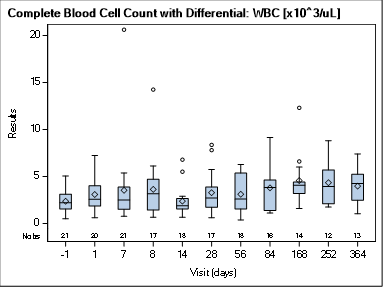

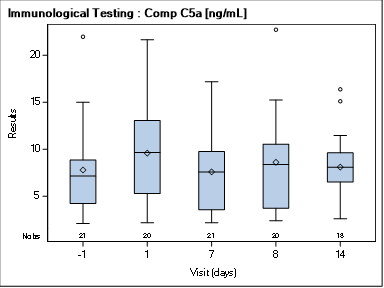
**

**Figure S9: Patient-level Display of anti-HLA Antibodies**


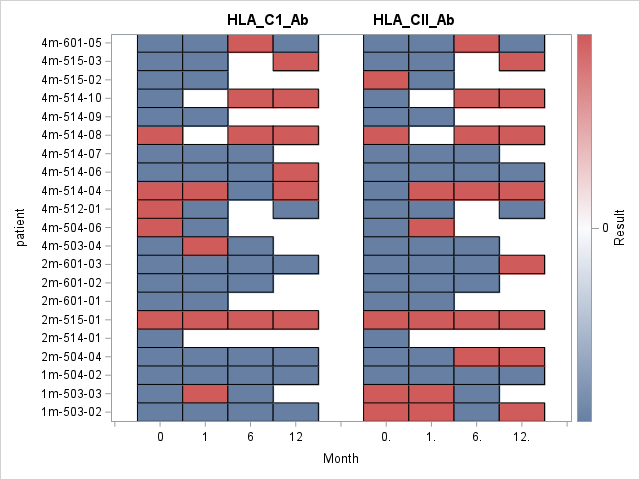


**Table S10: Modified IWG Criteria Used to Define Hematologic Response**

| **Blood Cell**  **Types** | **Baseline Levels** | **Hematologic Improvement Criteria** |
| --- | --- | --- |
| **Platelets** | **Platelets ≤ 20,000/µl** | Platelets ≥ 20,000/µL and platelets change from baseline ≥ platelets baseline (ie, platelets change from baseline ≥ 100% from baseline value) |
|  | **20,000/µl < platelets ≤ 100,000/µl** | Change from baseline ≥ 30,000/µL (absolute increase) |
|  | **Platelets > 100,000/ µl** | **Positive Platelets Responder** |
| **Erythroid (Hb)** | **Hb ≤ 9.0 g/dL** | Change from baseline ≥ 1.5 g/dL |
|  | **Hb > 9 g/dL** | **Positive Hb Responder** |
| **Neutrophiles (ANC)** | **ANC ≤ 500/µL** | ANC ≥ 500/µL and change from baseline ≥ ANC baseline (ie, ANC Change from baseline ≥ 100% from baseline value) |
|  | **500/µL <ANC ≤ 1000/µL** | ANC ≥ 1000/µL and change from baseline ≥ 0.5*ANC baseline (ie, ANC change from baseline ≥ 50% from baseline value) |
|  | **ANC > 1000/µL** | **Positive ANC Responder** |

To be considered a responder, patients had to a above the “responder” threshold at 6 months for at least 2 of the three lines. Patients entering the study with only one below the response threshold should be above the threshold for that cell line to be considered a responder.

**Table S11: Proportion of patients with a positive hematological response over time, baseline to month 6**

| **Visit** | **N** | **Mean** | **Std** |
| --- | --- | --- | --- |
| **Baseline** | 21 | 0 | 0 |
| **Day 1** | 21 | 0.19 | 0.40 |
| **Day 7** | 21 | 0.10 | 0.30 |
| **Day 8** | 20 | 0.20 | 0.41 |
| **Day 14** | 19 | 0.26 | 0.45 |
| **Month 1** | 20 | 0.45 | 0.51 |
| **Month 2** | 19 | 0.53 | 0.51 |
| **Month 3** | 19 | 0.53 | 0.51 |
| **Month 6** | 17 | 0.65 | 0.49 |

Std = standard deviation

**Table S12: Sub-group analysis of hematologic response at month 6, by selected parameters**

| **Subgroup** | **N** | **m-IWG** | | **PLT positive Response** | | **ANC positive Response** | | **HGB positive Response** | |
| --- | --- | --- | --- | --- | --- | --- | --- | --- | --- |
|  |  | **Mean** | **Std** | **Mean** | **Std** | **Mean** | **Std** | **Mean** | **Std** |
| **All** | 17 | 0.65 | 0.49 | 0.65 | 0.49 | 0.82 | 0.39 | 0.65 | 0.49 |
| **Platelets independent** | 7 | 0.86 | 0.38 | 0.86 | 0.38 | 1 | 0 | 0.86 | 0.38 |
| **Platelets dependent** | 10 | 0.5 | 0.53 | 0.5 | 0.53 | 0.7 | 0.48 | 0.5 | 0.53 |
| **RBC independent** | 7 | 0.71 | 0.49 | 0.71 | 0.49 | 0.86 | 0.38 | 0.71 | 0.49 |
| **RBC dependent** | 10 | 0.6 | 0.52 | 0.6 | 0.52 | 0.8 | 0.42 | 0.6 | 0.52 |
| **Female** | 6 | 0.5 | 0.55 | 0.5 | 0.55 | 0.67 | 0.52 | 0.5 | 0.55 |
| **Male** | 11 | 0.73 | 0.47 | 0.73 | 0.47 | 0.91 | 0.3 | 0.73 | 0.47 |
| **Age < med (56)** | 9 | 0.67 | 0.5 | 0.67 | 0.5 | 0.89 | 0.33 | 0.67 | 0.5 |
| **Age ≥ med (56)** | 8 | 0.63 | 0.52 | 0.63 | 0.52 | 0.75 | 0.46 | 0.63 | 0.52 |
| **GCSF_BS (Yes)** | 3 | 0.67 | 0.58 | 0.67 | 0.58 | 1 | 0 | 0.67 | 0.58 |
| **GCSF_BS (No)** | 14 | 0.64 | 0.5 | 0.64 | 0.5 | 0.79 | 0.43 | 0.64 | 0.5 |
| **GCSF_T180 (Yes)** | 8 | 0.63 | 0.52 | 0.63 | 0.52 | 0.75 | 0.46 | 0.63 | 0.52 |
| **GCSF_T180 (No)** | 9 | 0.67 | 0.5 | 0.67 | 0.5 | 0.89 | 0.33 | 0.67 | 0.5 |
| **GCSF_BS+180 (Yes)** | 8 | 0.63 | 0.52 | 0.63 | 0.52 | 0.75 | 0.46 | 0.63 | 0.52 |
| **GCSF_BS +180(No)** | 9 | 0.67 | 0.5 | 0.67 | 0.5 | 0.89 | 0.33 | 0.67 | 0.5 |
| **PS_BS (Yes)** | 4 | 0.75 | 0.5 | 0.75 | 0.5 | 1 | 0 | 0.75 | 0.5 |
| **PS_BS (No)** | 13 | 0.62 | 0.51 | 0.62 | 0.51 | 0.77 | 0.44 | 0.62 | 0.51 |
| **PS_T180 (Yes)** | 10 | 0.6 | 0.52 | 0.6 | 0.52 | 0.9 | 0.32 | 0.6 | 0.52 |
| **PS_T180 (No)** | 7 | 0.71 | 0.49 | 0.71 | 0.49 | 0.71 | 0.49 | 0.71 | 0.49 |
| **PS_BS+180 (Yes)** | 10 | 0.6 | 0.52 | 0.6 | 0.52 | 0.9 | 0.32 | 0.6 | 0.52 |
| **PS_BS +180(No)** | 7 | 0.71 | 0.49 | 0.71 | 0.49 | 0.71 | 0.49 | 0.71 | 0.49 |
| **Onset >240 days** | 7 | 0.43 | 0.53 | 0.43 | 0.53 | 0.86 | 0.38 | 0.43 | 0.53 |
| **Onset <=240 days** | 10 | 0.80 | 0.42 | 0.80 | 0.42 | 0.80 | 0.42 | 0.80 | 0.42 |

ANC=absolute neutrophil count; BS=baseline; GCSF=granulocyte colony stimulating factor (or similar agents); HGB=hemoglobin; Med=median; PLT=platelets; PS=platelet stimulating factors (TPO receptor agonists); RBC=red blood cells.

Onset=time from HCT to study drug administration; T180=study day 180; BS+180= at anytime from baseline to day 180

**Figure S13: Patient level presentation of blood counts, use of blood product transfusions and hematopoietic growth factors**

Patient Level Presentation of Absolute Neutrophil Count and G-CSF Consumption for Patients with HCT Onset ≥ 270 days and Patients with HCT Onset < 270 days


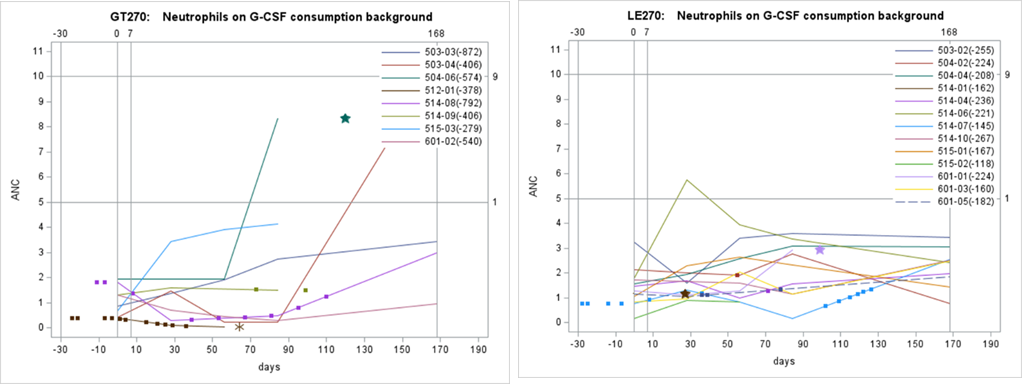


ANC=absolute neutrophil count; G-CSF=granulocyte colony stimulating factor; squares= time of colony stimulating factors consumption, stars = time of death, asterisk = time of early termination.

The number of days between HCT and PLX-R18 treatment appears for each patient in parentheses after his/her patient number. The left panel presents patients with more than 270 days between the two events (GT270); the right panel presents patients with 270 days or less between the two events (LE270).

Patient Level Presentation of Hemoglobin Levels with Red Blood Cell Transfusions and Administration of Erythropoietin Stimulating Agents for Patients with HCT Onset ≥ 270 days and Patients with HCT Onset < 270 days

EPO=erythropoietin stimulating agents; HGB=hemoglobin; RBC=red blood cell; circles= time of RBC transfusions; squares= time of EPO consumption, stars = time of death, asterisk = time of early termination.


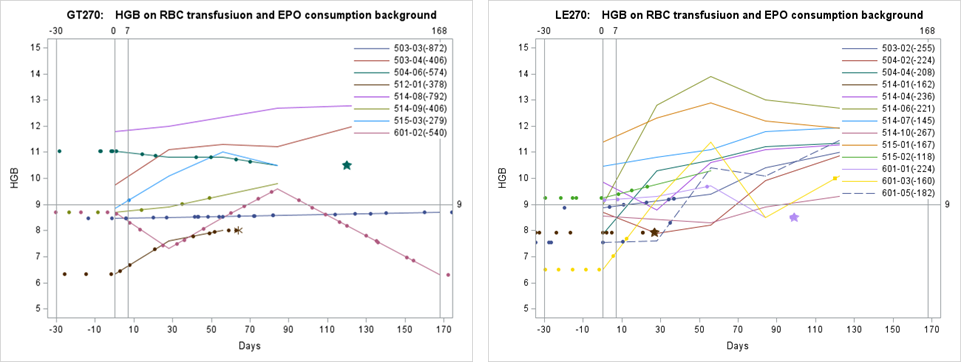
Note: The number of days between HCT and PLX-R18 treatment appears for each patient in parentheses after his/her patient number. The left panel presents patients with more than 270 days between the two events (GT270); the right panel presents patients with 270 days or less between the two events (LE270).

Patient Level Presentation of Platelet Counts with Platelet Transfusions and Administration of Platelet Stimulants for Patients with HCT Onset ≥ 270 days and Patients with HCT Onset < 270 days

**
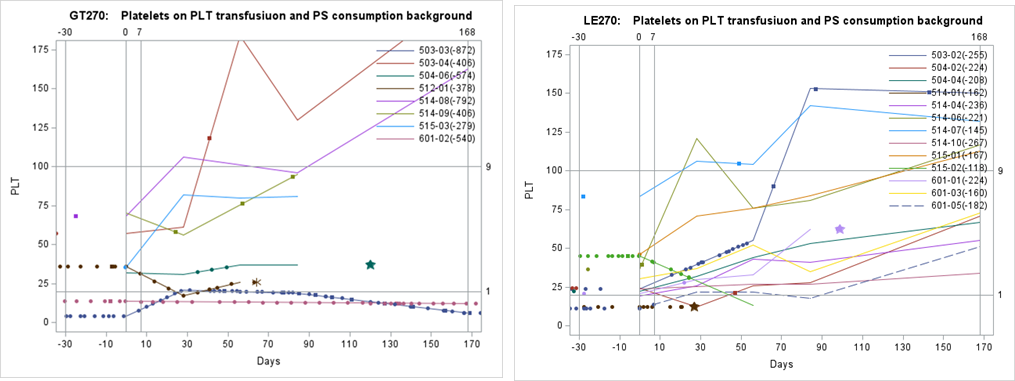
**

PLT=platelets; PS=platelet stimulating agents; circles= time of PLT transfusions; squares= time of PS consumption, stars = time of death, asterisk = time of early termination.

Note: The number of days between HCT and PLX-R18 treatment appears for each patient in parentheses after his/her patient number. The left panel presents patients with more than 270 days between the two events (GT270); the right panel presents patients with 270 days or less between the two events (LE270).

**Figure S14: SF-36V2 Quality of life scores over time by treatment cohort**

| Parameter | Treatment | Visit (days) | N | Mean | Median | Std | Min | Max |
| --- | --- | --- | --- | --- | --- | --- | --- | --- |
| Emotional well-being | PLX-R18 1M cells/kg | 0 | 3 | 76.7 | 65.0 | 20.2 | 65.0 | 100.0 |
|  |  | 28 | 3 | 85.0 | 80.0 | 13.2 | 75.0 | 100.0 |
|  |  | 84 | 3 | 78.3 | 85.0 | 16.1 | 60.0 | 90.0 |
|  |  | 168 | 3 | 80.0 | 80.0 | 10.0 | 70.0 | 90.0 |
|  |  | 364 | 2 | 82.5 | 82.5 | 10.6 | 75.0 | 90.0 |
|  | PLX-R18 2M cells/kg | 0 | 6 | 82.5 | 85.0 | 4.2 | 75.0 | 85.0 |
|  |  | 28 | 5 | 78.0 | 80.0 | 7.6 | 65.0 | 85.0 |
|  |  | 84 | 5 | 71.0 | 85.0 | 20.4 | 40.0 | 85.0 |
|  |  | 168 | 4 | 76.3 | 82.5 | 14.4 | 55.0 | 85.0 |
|  |  | 364 | 4 | 77.5 | 85.0 | 26.0 | 40.0 | 100.0 |
|  | PLX-R18 4M cells/kg | 0 | 10 | 70.5 | 65.0 | 19.4 | 50.0 | 100.0 |
|  |  | 28 | 8 | 63.1 | 60.0 | 16.2 | 50.0 | 100.0 |
|  |  | 84 | 6 | 62.5 | 60.0 | 26.2 | 35.0 | 100.0 |
|  |  | 168 | 3 | 83.3 | 80.0 | 10.4 | 75.0 | 95.0 |
|  |  | 364 | 2 | 85.0 | 85.0 | 21.2 | 70.0 | 100.0 |
| Energy/fatigue | PLX-R18 1M cells/kg | 0 | 3 | 41.7 | 56.3 | 30.8 | 6.3 | 62.5 |
|  |  | 28 | 3 | 25.0 | 25.0 | 6.3 | 18.8 | 31.3 |
|  |  | 84 | 3 | 45.8 | 50.0 | 7.2 | 37.5 | 50.0 |
|  |  | 168 | 3 | 43.8 | 43.8 | 6.3 | 37.5 | 50.0 |
|  |  | 364 | 2 | 40.6 | 40.6 | 13.3 | 31.3 | 50.0 |
|  | PLX-R18 2M cells/kg | 0 | 6 | 55.2 | 59.4 | 19.9 | 31.3 | 75.0 |
|  |  | 28 | 5 | 66.3 | 75.0 | 24.4 | 37.5 | 93.8 |
|  |  | 84 | 5 | 55.0 | 50.0 | 10.3 | 43.8 | 68.8 |
|  |  | 168 | 4 | 54.7 | 56.3 | 12.9 | 37.5 | 68.8 |
|  |  | 364 | 4 | 57.8 | 56.3 | 28.1 | 25.0 | 93.8 |
|  | PLX-R18 4M cells/kg | 0 | 10 | 43.8 | 46.9 | 22.0 | 12.5 | 81.3 |
|  |  | 28 | 8 | 45.3 | 50.0 | 28.5 | 0.0 | 81.3 |
|  |  | 84 | 6 | 47.9 | 50.0 | 21.9 | 12.5 | 75.0 |
|  |  | 168 | 3 | 60.4 | 56.3 | 19.1 | 43.8 | 81.3 |
|  |  | 364 | 2 | 53.1 | 53.1 | 4.4 | 50.0 | 56.3 |
| General health | PLX-R18 1M cells/kg | 0 | 3 | 40.0 | 45.0 | 22.9 | 15.0 | 60.0 |
|  |  | 28 | 3 | 40.0 | 40.0 | 10.0 | 30.0 | 50.0 |
|  |  | 84 | 3 | 51.7 | 50.0 | 22.5 | 30.0 | 75.0 |
|  |  | 168 | 3 | 51.7 | 55.0 | 10.4 | 40.0 | 60.0 |
|  |  | 364 | 2 | 47.5 | 47.5 | 3.5 | 45.0 | 50.0 |
|  | PLX-R18 2M cells/kg | 0 | 6 | 48.3 | 50.0 | 5.2 | 40.0 | 55.0 |
|  |  | 28 | 5 | 49.0 | 50.0 | 24.3 | 20.0 | 85.0 |
|  |  | 84 | 5 | 44.0 | 30.0 | 23.3 | 25.0 | 80.0 |
|  |  | 168 | 4 | 45.0 | 45.0 | 12.9 | 30.0 | 60.0 |
|  |  | 364 | 4 | 46.3 | 42.5 | 21.7 | 25.0 | 75.0 |
|  | PLX-R18 4M cells/kg | 0 | 11 | 47.7 | 50.0 | 20.2 | 15.0 | 70.0 |
|  |  | 28 | 8 | 51.3 | 42.5 | 21.2 | 25.0 | 85.0 |
|  |  | 84 | 6 | 54.2 | 50.0 | 21.8 | 30.0 | 85.0 |
|  |  | 168 | 3 | 58.3 | 45.0 | 27.5 | 40.0 | 90.0 |
|  |  | 364 | 2 | 37.5 | 37.5 | 3.5 | 35.0 | 40.0 |
| Pain | PLX-R18 1M cells/kg | 0 | 3 | 64.2 | 67.5 | 8.0 | 55.0 | 70.0 |
|  |  | 28 | 3 | 63.3 | 67.5 | 16.6 | 45.0 | 77.5 |
|  |  | 84 | 3 | 75.0 | 77.5 | 16.4 | 57.5 | 90.0 |
|  |  | 168 | 3 | 60.0 | 67.5 | 13.0 | 45.0 | 67.5 |
|  |  | 364 | 2 | 51.3 | 51.3 | 8.8 | 45.0 | 57.5 |
|  | PLX-R18 2M cells/kg | 0 | 6 | 65.0 | 73.8 | 38.0 | 10.0 | 100.0 |
|  |  | 28 | 5 | 68.5 | 87.5 | 39.7 | 10.0 | 100.0 |
|  |  | 84 | 5 | 56.5 | 47.5 | 29.1 | 22.5 | 100.0 |
|  |  | 168 | 4 | 50.0 | 43.8 | 29.9 | 22.5 | 90.0 |
|  |  | 364 | 4 | 60.6 | 60.0 | 31.9 | 22.5 | 100.0 |
|  | PLX-R18 4M cells/kg | 0 | 10 | 80.3 | 85.0 | 25.0 | 22.5 | 100.0 |
|  |  | 28 | 8 | 86.9 | 95.0 | 16.8 | 57.5 | 100.0 |
|  |  | 84 | 6 | 74.2 | 88.8 | 38.9 | 0.0 | 100.0 |
|  |  | 168 | 3 | 70.0 | 77.5 | 34.4 | 32.5 | 100.0 |
|  |  | 364 | 2 | 50.0 | 50.0 | 38.9 | 22.5 | 77.5 |
| Physical functioning | PLX-R18 1M cells/kg | 0 | 3 | 50.0 | 55.0 | 37.7 | 10.0 | 85.0 |
|  |  | 28 | 3 | 33.3 | 40.0 | 20.8 | 10.0 | 50.0 |
|  |  | 84 | 3 | 56.7 | 65.0 | 23.6 | 30.0 | 75.0 |
|  |  | 168 | 3 | 58.3 | 50.0 | 14.4 | 50.0 | 75.0 |
|  |  | 364 | 2 | 62.5 | 62.5 | 10.6 | 55.0 | 70.0 |
|  | PLX-R18 2M cells/kg | 0 | 6 | 45.8 | 40.0 | 20.8 | 25.0 | 80.0 |
|  |  | 28 | 5 | 44.0 | 35.0 | 32.5 | 0.0 | 85.0 |
|  |  | 84 | 5 | 48.0 | 40.0 | 23.6 | 25.0 | 80.0 |
|  |  | 168 | 4 | 41.3 | 35.0 | 16.0 | 30.0 | 65.0 |
|  |  | 364 | 4 | 25.0 | 25.0 | 8.2 | 15.0 | 35.0 |
|  | PLX-R18 4M cells/kg | 0 | 10 | 57.5 | 57.5 | 27.7 | 20.0 | 95.0 |
|  |  | 28 | 8 | 59.4 | 65.0 | 27.4 | 15.0 | 90.0 |
|  |  | 84 | 6 | 61.7 | 70.0 | 37.8 | 15.0 | 95.0 |
|  |  | 168 | 3 | 68.3 | 70.0 | 27.5 | 40.0 | 95.0 |
|  |  | 364 | 2 | 50.0 | 50.0 | 35.4 | 25.0 | 75.0 |
| Role limit. due to emotional problems | PLX-R18 1M cells/kg | 0 | 3 | 69.4 | 83.3 | 39.4 | 25.0 | 100.0 |
|  |  | 28 | 3 | 66.7 | 75.0 | 38.2 | 25.0 | 100.0 |
|  |  | 84 | 3 | 83.3 | 75.0 | 14.4 | 75.0 | 100.0 |
|  |  | 168 | 3 | 83.3 | 100.0 | 28.9 | 50.0 | 100.0 |
|  |  | 364 | 2 | 70.8 | 70.8 | 41.2 | 41.7 | 100.0 |
|  | PLX-R18 2M cells/kg | 0 | 6 | 66.7 | 79.2 | 39.8 | 8.3 | 100.0 |
|  |  | 28 | 5 | 71.7 | 100.0 | 41.5 | 8.3 | 100.0 |
|  |  | 84 | 5 | 88.3 | 100.0 | 21.7 | 50.0 | 100.0 |
|  |  | 168 | 4 | 83.3 | 91.7 | 23.6 | 50.0 | 100.0 |
|  |  | 364 | 4 | 83.3 | 91.7 | 23.6 | 50.0 | 100.0 |
|  | PLX-R18 4M cells/kg | 0 | 10 | 72.5 | 91.7 | 36.0 | 16.7 | 100.0 |
|  |  | 28 | 8 | 67.7 | 70.8 | 26.5 | 16.7 | 100.0 |
|  |  | 84 | 6 | 70.8 | 75.0 | 31.1 | 25.0 | 100.0 |
|  |  | 168 | 3 | 91.7 | 100.0 | 14.4 | 75.0 | 100.0 |
|  |  | 364 | 2 | 75.0 | 75.0 | 35.4 | 50.0 | 100.0 |
| Role limitations due to physical health | PLX-R18 1M cells/kg | 0 | 3 | 41.7 | 56.3 | 36.6 | 0.0 | 68.8 |
|  |  | 28 | 3 | 39.6 | 43.8 | 37.7 | 0.0 | 75.0 |
|  |  | 84 | 3 | 68.8 | 62.5 | 28.6 | 43.8 | 100.0 |
|  |  | 168 | 3 | 64.6 | 50.0 | 30.8 | 43.8 | 100.0 |
|  |  | 364 | 2 | 43.8 | 43.8 | 8.8 | 37.5 | 50.0 |
|  | PLX-R18 2M cells/kg | 0 | 6 | 19.8 | 21.9 | 17.4 | 0.0 | 43.8 |
|  |  | 28 | 5 | 37.5 | 50.0 | 20.3 | 12.5 | 56.3 |
|  |  | 84 | 5 | 33.8 | 50.0 | 28.5 | 0.0 | 62.5 |
|  |  | 168 | 4 | 51.6 | 46.9 | 31.2 | 18.8 | 93.8 |
|  |  | 364 | 4 | 43.8 | 34.4 | 41.8 | 6.3 | 100.0 |
|  | PLX-R18 4M cells/kg | 0 | 10 | 46.3 | 46.9 | 32.6 | 0.0 | 93.8 |
|  |  | 28 | 8 | 46.1 | 43.8 | 21.4 | 6.3 | 75.0 |
|  |  | 84 | 6 | 60.4 | 65.6 | 33.0 | 18.8 | 93.8 |
|  |  | 168 | 3 | 75.0 | 75.0 | 25.0 | 50.0 | 100.0 |
|  |  | 364 | 2 | 40.6 | 40.6 | 13.3 | 31.3 | 50.0 |
| Social functioning | PLX-R18 1M cells/kg | 0 | 3 | 33.3 | 25.0 | 38.2 | 0.0 | 75.0 |
|  |  | 28 | 3 | 45.8 | 37.5 | 26.0 | 25.0 | 75.0 |
|  |  | 84 | 3 | 58.3 | 50.0 | 14.4 | 50.0 | 75.0 |
|  |  | 168 | 3 | 75.0 | 87.5 | 21.7 | 50.0 | 87.5 |
|  |  | 364 | 2 | 56.3 | 56.3 | 26.5 | 37.5 | 75.0 |
|  | PLX-R18 2M cells/kg | 0 | 6 | 60.4 | 50.0 | 26.7 | 37.5 | 100.0 |
|  |  | 28 | 5 | 60.0 | 50.0 | 38.9 | 12.5 | 100.0 |
|  |  | 84 | 5 | 57.5 | 50.0 | 30.1 | 25.0 | 100.0 |
|  |  | 168 | 4 | 53.1 | 43.8 | 32.9 | 25.0 | 100.0 |
|  |  | 364 | 4 | 59.4 | 56.3 | 40.0 | 25.0 | 100.0 |
|  | PLX-R18 4M cells/kg | 0 | 10 | 63.8 | 75.0 | 36.1 | 0.0 | 100.0 |
|  |  | 28 | 8 | 65.6 | 68.8 | 23.9 | 25.0 | 100.0 |
|  |  | 84 | 6 | 50.0 | 56.3 | 20.9 | 25.0 | 75.0 |
|  |  | 168 | 3 | 83.3 | 87.5 | 7.2 | 75.0 | 87.5 |
|  |  | 364 | 2 | 75.0 | 75.0 | 35.4 | 50.0 | 100.0 |

Std=standard deviation, Max=maximum; Min=minimum

**Table S15: SF-36V2 Quality of life scores over time for the overall population**

| PARAMETER | Visit (days) | N | Mean | Std | p-value |
| --- | --- | --- | --- | --- | --- |
| **Emotional well-being** | 28 | 15 | -2.3 | 12.1 | 0.467 |
|  | 84 | 14 | -6.4 | 14.9 | 0.130 |
|  | 168 | 10 | 3.5 | 15.6 | 0.497 |
|  | 364 | 8 | -0.6 | 16.4 | 0.917 |
| **Energy/fatigue** | 28 | 15 | -0.8 | 18.3 | 0.862 |
|  | 84 | 14 | 3.1 | 19.9 | 0.566 |
|  | 168 | 10 | 4.4 | 19.1 | 0.487 |
|  | 364 | 8 | 3.1 | 22.9 | 0.711 |
| **General health** | 28 | 15 | -0.3 | 16.5 | 0.939 |
|  | 84 | 14 | 2.9 | 16.8 | 0.537 |
|  | 168 | 10 | 4.5 | 15.5 | 0.384 |
|  | 364 | 8 | -6.3 | 17.5 | 0.345 |
| **Pain** | 28 | 15 | 3.7 | 19.8 | 0.485 |
|  | 84 | 14 | -2.3 | 30.0 | 0.777 |
|  | 168 | 10 | -2.5 | 24.7 | 0.756 |
|  | 364 | 8 | -7.5 | 39.9 | 0.611 |
| **Physical functioning** | 28 | 15 | -2.3 | 17.4 | 0.612 |
|  | 84 | 14 | 5.4 | 17.4 | 0.269 |
|  | 168 | 10 | 5.0 | 22.2 | 0.495 |
|  | 364 | 8 | -8.1 | 24.3 | 0.377 |
| **Role limit. due to emotional problems** | 28 | 15 | 0.0 | 30.0 | 1.000 |
|  | 84 | 14 | 9.5 | 27.3 | 0.215 |
|  | 168 | 10 | 18.3 | 37.6 | 0.158 |
|  | 364 | 8 | 5.2 | 32.7 | 0.666 |
| **Role limitations due to physical health** | 28 | 15 | 7.1 | 17.7 | 0.143 |
|  | 84 | 14 | 15.2 | 23.0 | 0.028 |
|  | 168 | 10 | 29.4 | 23.4 | 0.003 |
|  | 364 | 8 | 12.5 | 29.3 | 0.267 |
| **Social functioning** | 28 | 15 | 8.3 | 21.5 | 0.155 |
|  | 84 | 14 | 0.0 | 29.4 | 1.000 |
|  | 168 | 10 | 16.3 | 33.9 | 0.164 |
|  | 364 | 8 | 7.8 | 24.9 | 0.405 |

Std=standard deviation, Max=maximum; Min=minimum

**Table S16: SF-36V2 Quality of life analysis by physical and mental component summary scales**

| **Parameter** | **level** | **PLX-R18 1M** | **PLX-R18 2M** | **PLX-R18 4M** | **Total** | **P-Value** |
| --- | --- | --- | --- | --- | --- | --- |
| **MONTH 6 CHANGE FROM BASELINE PCS** | Ns | 3 | 4 | 3 | 10 | 0.3503 |
|  | Mean(Std) | 4.71 (2.99) | 4.16 (5.29) | -0.61 (5.00) | 2.89 (4.77) |  |
|  | Median | 4.7 | 4.8 | 0 | 2.9 |  |
|  | Min, Max | 1.72 , 7.70 | -2.07 , 9.08 | -5.90 , 4.04 | -5.90 , 9.08 |  |
| **MONTH 6 CHANGE FROM BASELINE MCS** | Ns | 3 | 4 | 3 | 10 | 0.4151 |
|  | Mean(Std) | 5.70 (16.43) | -1.88 (8.87) | 10.78 (10.47) | 4.19 (11.92) |  |
|  | Median | 0.5 | -1.1 | 14.4 | 0.5 |  |
|  | Min, Max | -7.51 , 24.10 | -13.31 , 8.06 | -1.02 , 18.97 | -13.31 , 24.10 |  |

Std=standard deviation, Max=maximum; Min=minimum; PCS=physical component summary measure; MCS=mental component summary scales.

Data is presented for the subset of patients for which baseline and month 6 SF-36V2 results are available (n=10). PCS and MCS were calculated according to methodology presented in: Ware JE, Kosinski M, & Keller SD. *SF-36 Physical and Mental Health Summary Scales: A User’s Manual*. Boston, MA: Health Assessment Lab, 1994.

**Table S17: List of ethics committees/institutional review boards**

| Site number | EC name | EC address |
| --- | --- | --- |
| 502 | Memorial Sloan Kettering | Memorial Sloan-Kettering Cancer Center  1275 York Avenue, New York, New York 10065 |
| 503 | University Hospitals Cleveland Medical Center  Institutional REview Board for Human Investigation | 11100 Euclid Ave  Cleveland, HO 44106 |
| 504 | Baylor Scott & White Research Institute | 3310 Live Oak  Suite 501  Dallas, TX 75204 |
| 510 | University of Maryland - Baltimore  Institutional Review Board | 620 West Lexington St, Second Floor  Baltimore, MD 21201 |
| 511 | BSD IRB Committee A  The University of Chicago Biological Sciences Division/University of Chicago  Medical Center | 5841 S. Maryland Ave.,  MC7132, I-625  Chicago, IL 60637 |
| 512 | uses WIRB as the local IRB, completes all submissions on their own.  Western IRB | 1019 39th Ave SE #120  Puyallup, WA 98374 |
| 514 | The University of Kansas Medical Center  Human Research Protection Program | Mail-Stop 1032  3901 Rainbow Blvd.  Kansas City, KS 66160 |
| 515 | WIRB as the central IRB  Western IRB | 1019 39th Ave SE #120  Puyallup, WA 98374 |
| 516 | University of Miami  Human Subject Research Office (M809) | 1400 NW 10th Avenue, Suite 1200A  Miami, FL 33136 |
| 601 | IRB Rambam | HaAliya HaShniya St 8, Haifa,IL 3109601 |
| 602 | IRB Hadassah Ein Kerem | Kiryat Hadassah  POB 12000, Jerusalem,IL 91120 |
